# Noisy models of the ventral stream reveal the impact of recurrence and learned representations on information processing timescales

**DOI:** 10.1371/journal.pcbi.1014653

**Published:** 2026-08-27

**Authors:** Sara Varetti, Sebastian Goldt, Eugenio Piasini

**Affiliations:** 1 Cognitive Neuroscience, International School for Advanced Studies (SISSA), Trieste, Italy; 2 Theoretical and Scientific Data Science, International School for Advanced Studies (SISSA), Trieste, Italy; ShanghaiTech University, CHINA

## Abstract

In vision neuroscience, the temporal dynamics of the sensory stream and of its neural representations are thought to be deeply linked to the function of the hierarchy of cortical areas that deal with object recognition, known as the visual ventral stream. Neural representations that are invariant under identity-preserving object transformations, and therefore allow for efficient learning of object identity, are theorized to emerge from a self-supervised learning process that attempts to extract “temporally stable” features from the sensory input. Conversely, invariance increases along the hierarchy, putatively implying progressively slower neural codes in higher-level areas. Recent neurophysiological evidence shows that indeed, as one moves along this cortical hierarchy, neural representations of dynamic stimuli become slower, and additionally the temporal scales of the within-trial fluctuations of these representations (called “intrinsic timescales”) increase starkly. However, while these timescale hierarchies have been reproduced in biologically grounded recurrent models, their network determinants have remained largely unexplored in image-computable models of the ventral stream. Here we investigate the temporal structure of the neural codes in a noisy, recurrent and adaptive model of the ventral visual stream. We show that, surprisingly, the organization of the representation timescales is set by the broad architectural features of the network, regardless of training, while the ordering of the intrinsic timescales across layers is sensitive to the details of the functions implemented by each layer. Our work underscores the importance of the temporal structure of the neural code as a probe for the link between structure and function in models of the vertebrate visual system.

## Author summary

Making sense of a constantly changing visual world requires the brain to integrate information over time. As visual signals travel through the hierarchy of cortical areas that support object recognition, neural representations are thought to become progressively more stable in time. Recent experiments have confirmed this, showing that both the timescales of stimulus-driven responses and those of the fluctuations around average responses (the “intrinsic timescales”) grow along the hierarchy. Yet the artificial neural networks most commonly used to model vision are static and cannot capture these dynamics. Here we built a family of biologically inspired convolutional–recurrent networks that process movies while incorporating noise, recurrence, and adaptation. By introducing these ingredients one at a time, we asked which are actually needed to reproduce the experimentally observed hierarchy of timescales. We found that the ordering of response timescales depends chiefly on the broad architecture of the network, even in untrained (random) networks. In contrast, the hierarchy of intrinsic timescales is far more fragile: it requires both slow internal dynamics and representations shaped by learning. Our results suggest that intrinsic fluctuations are an informative and underused benchmark for computational models of the visual system.

## Introduction

A major goal in vision neuroscience is to build models that capture the essential computational principles by which the visual cortex processes real-world inputs. In the last decade, convolutional neural networks (CNNs) have been found to be remarkably successful at modeling visual recognition from static images. This class of artificial neural networks has also demonstrated a remarkable ability to approximate cortical representations [1,2], revealing hierarchical processing of inputs [3] and shedding light on the functional computations performed by the biological visual cortex [4,5]. Despite their success in modeling cortical responses to static images, popular CNN-based models of visual cortex do not attempt to capture the inherently dynamic nature of visual experience, where information is continuously integrated across spatial [6,7] and temporal [8] dimensions. After early attempts to use recurrence to improve the processing of static images [9] and theoretical insight that linked the depth advantage of certain residual networks to their mimicking of recurrent mechanisms [10], recently several architectures combining aspects of CNNs and recurrent networks (CNN-RNNs) have been proposed to bridge the gap between the spatial processing capabilities of convolutional networks and the need for temporal integration mechanisms. These architectures typically rely on recurrent connections to capture dynamic aspects of visual input [11–14]. Recurrent components are also included by large-scale efforts to build “foundation models” for visual neuroscience [15]. However, little attention has been devoted so far to the systematic study of the temporal properties of modeled activity. In particular, experimental evidence has shown that neural activity timescales are organized hierarchically, both within sensory cortex and across the brain more broadly, in humans, nonhuman primates, and rodents [8,16–21]. An increase in the timescale, or the temporal stability, of neural codes is a crucial feature of classical and modern accounts of the emergence of invariance in sensory cortices [22–30]. Therefore, it is natural to ask under what conditions our models of visual cortex possess a similar property. To address this question, in this work we design and study a hybrid architecture that integrates convolutions, recurrent dynamics, adaptation and intrinsic noise, while preserving a simple structure that allows us to develop architectural parallels to visual cortex. The model is built on top of CORnet [14,31] and from that architecture inherits a small number of layers that can be directly mapped onto areas of the ventral visual stream, enabling a biologically grounded investigation of temporal processing. We focus our investigation on two notions of timescales, as formalized by [19]: the timescale of the average neural representation of a stimulus (*response timescale*), and the timescale of random fluctuations around the average (*intrinsic timescale*). Our analyses show that, while a hierarchy of response timescales emerge robustly as a byproduct of the large-scale organization of network models, the organization of intrinsic timescales is sensitive to the fine details of a model and of its training. These results lead us to argue that intrinsic timescales are a useful conceptual construct that could guide the design and development of computational models of sensory systems.

## Results

**Fig 1:**
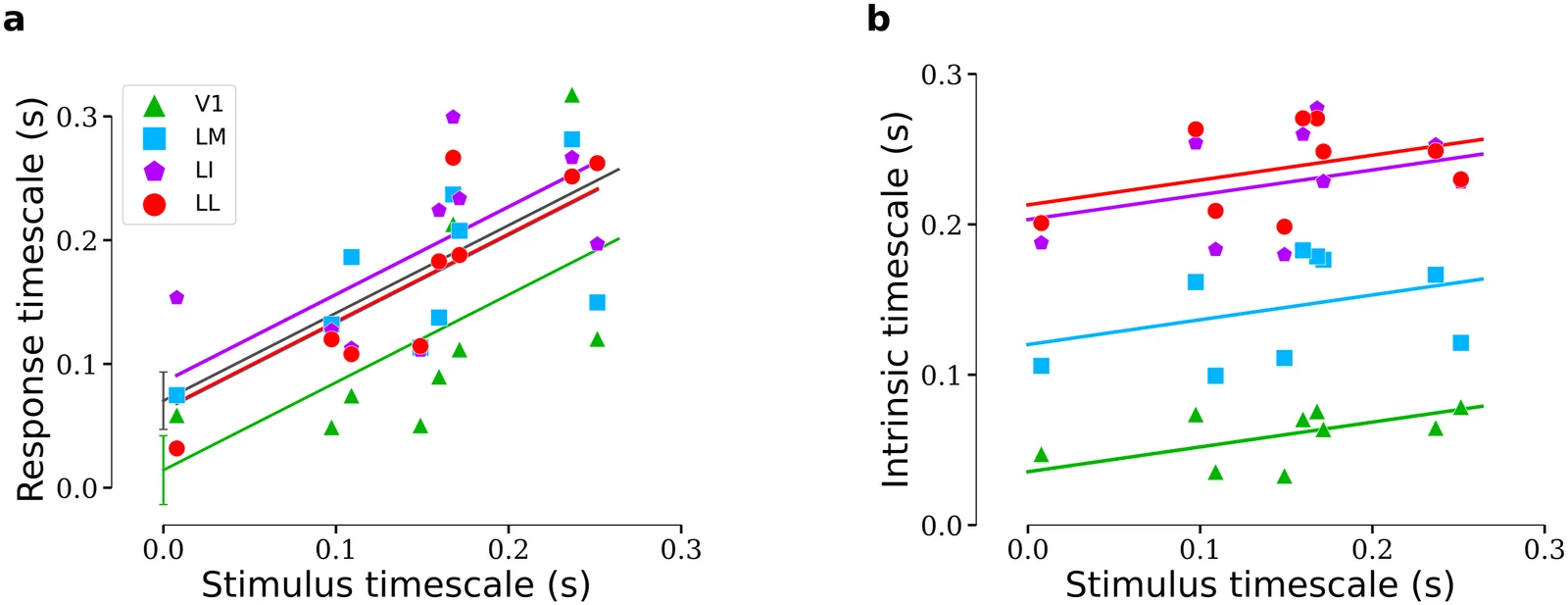
Response and intrinsic timescales in rat visual cortex, from [19]. Response a) and intrinsic timescales b) as a function of stimulus timescales for four visual areas (V1, LM, LI, LL). The stimulus timescale represents the decay time of the pixel-wise correlation between frames in each dynamic input (movie), i.e., how rapidly the visual content changes over time. Response, intrinsic, and stimulus timescales were estimated from neuronal recordings during presentation of naturalistic movie stimuli (see Methods for details on the computation of autocorrelation functions and the fitting procedure used to extract timescales).

To quantify the timescales of neural codes for visual stimuli, we take inspiration from the empirical observations in [19], where electrophysiological recordings were made in rat visual cortex while the animal was exposed to dynamic visual stimuli (natural and synthetic videos) Fig 1. More specifically, data was collected from four cortical areas that constitute the rat analogue of the ventral visual stream — striate (V1), lateromedial (LM), laterointermediate (LI), and laterolateral (LL) visual cortex [32–34]. In [19], the temporal structure of the neural responses were characterized by two distinct measures: response timescales and intrinsic timescales. The response timescale captures the dynamics of the average neural response to a certain stimulus, and is expected to increase along the ventral stream by virtue of an increase in invariance of neural representations under identity-preserving transformations [27]. It can also be seen as the characteristic timescale of the *signal correlations* within the system, if the instantaneous inputs at two different time points are taken to be the analogues of distinct stimuli in the classical view [35,36]. The intrinsic timescale depends only on the trial-by-trial fluctuations of the neural responses around the average (see Estimation of Temporal Correlation in Methods for more details); it is, in other words, the timescale of temporal noise correlations [18,37,38], and as such it has been proposed to be related to the role of recurrent and adaptive mechanisms in neural circuits [19]. These timescale measures converge on the same quantity (albeit with differences in their sampling properties) for an homogeneous, stationary system, such as a population of identical neurons that are undergoing spontaneous activity. However, unlike other common measures based on spike count autocorrelations (e.g., [17]) they allow for a finer analysis of systems that process a time-varying stimulus that is repeated a number of times, which is a highly relevant setup when investigating sensory processing in cortical circuits.

Our goal in the present work is to identify the minimal set of mechanisms that a CNN-RNN architecture requires to reproduce this hierarchical organization of response and intrinsic timescales across cortical areas. To directly test which architectural elements are sufficient to reproduce these dynamics, we construct a family of CNN-RNN models capable of processing the same visual stimuli shown to the rats in the experiments. We build upon CORnet [14,31] as a baseline model of the ventral stream and introduce a series of minimal but systematic modifications. We feed the movies to each model and measure the resulting timescales, yielding results that are directly comparable to those in [19] (note that CORnet is designed to model neural activity in primates, and not in rat, but as mentioned above the hierarchical organization of timescales across cortical areas is a general feature of neural activity shared by rodents and primates).

A summary of the timescale dynamics resulting from each architectural modification is provided in Table 1. This stepwise approach allowed us to isolate how each component contributes to stable and temporally persistent activity patterns across the hierarchy of areas of the network.

**Table 1:** Summary of model variants and their effect on the presence of a hierarchy of response and intrinsic timescales, as well as on image recognition performance. Each model builds incrementally on the original CORnet architecture with minimal modifications. Presence/absence of a temporal hierarchy is determined from fitted autocorrelation functions. Variables: $h_{t}^{\left(l\right)}$ (hidden state at layer *l*, time *t*); $x_{t}^{\left(l-1\right)}$ (input from previous layer); $s_{t}^{\left(l\right)}$ (adaptation variable); γ (leak); β (adaptation strength); $\eta_{t}^{\left(l\right)}$ (noise).

Model variant	Top-1 (%)	Response	Intrinsic	Update equation
Original	55.40±0.08	×	—	$h_{t}^{\left(l\right)}=W(x_{t}^{\left(l-1\right)}+r_{t-1}^{\left(l\right)})$
Stochastic	54.23±0.07	×	×	$h_{t}^{\left(l\right)}=W(x_{t}^{\left(l-1\right)}+r_{t-1}^{\left(l\right)})+\eta_{t}^{\left(l\right)}$
Leaky	32.38±0.03	×	×	$h_{t}^{\left(l\right)}=W(x_{t}^{\left(l-1\right)}+r_{t-1}^{\left(l\right)})+{(1-\gamma )h}_{t-1}^{\left(l\right)}+\eta_{t}^{\left(l\right)}$
Adaptive	37.35±0.04	✓	✓	$h_{t}^{\left(l\right)}=W(x_{t}^{\left(l-1\right)}-{\beta s}_{t}^{\left(l\right)})+(1-\gamma )h_{t-1}^{\left(l\right)}+\eta_{t}^{\left(l\right)}$
Minimal	37.28±0.03	✓	✓	$h_{t}^{\left(l\right)}=W(x_{t}^{\left(l-1\right)})+(1-\gamma )h_{t-1}^{\left(l\right)}+\eta_{t}^{\left(l\right)}$
Random	2.35±0.47	✓	×	$h_{t}^{\left(l\right)}=W(x_{t}^{\left(l-1\right)}+r_{t}^{\left(l\right)})+\eta_{t}^{\left(l\right)}$
MLP	—	✓	×	$h_{t}^{\left(l\right)}=W(x_{t}^{\left(l-1\right)})+(1-\gamma )h_{t-1}^{\left(l\right)}+\eta_{t}$

**Table notes:** Presence/absence of a hierarchy is determined from the fitted autocorrelation functions (see Methods for details on estimation and fitting).

### Stochastic CORnet

**Fig 2:**
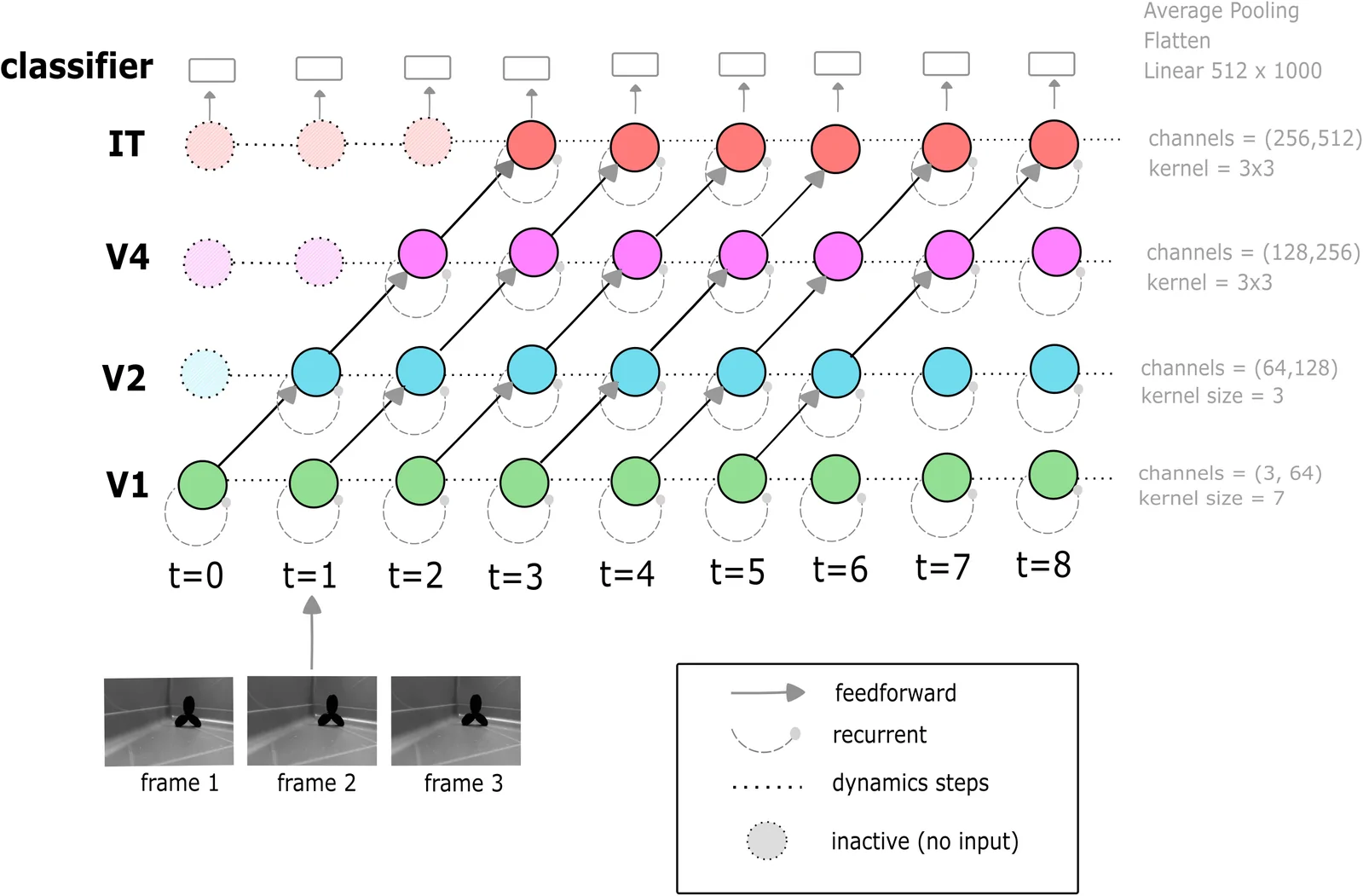
Network with recurrent and feedforward connections unrolled in biological time, introduced for static input in [14]. At each timestep, a new video frame is fed into V1, while the activity of higher visual areas (V2, V4, IT) evolves with a one-step delay relative to the previous layer. This staggered propagation causes information to flow sequentially through the hierarchy, so that at t = 0 only V1 is driven by the input, at t = 1 V2 becomes active, and so on, until the signal reaches IT after several timesteps. Dashed circles indicate the internal recurrent dynamics, while solid arrows mark the feedforward drive linking successive areas. This temporally unrolled scheme differs from conventional recurrent implementations, where all layers are updated simultaneously, and instead captures a more biologically realistic cascade of activation across the ventral stream. See S5 Fig for an expanded schematic showing more in detail the computations carried out by V1 and V2 over two timesteps for one of our network variants. Video frame images adapted from [19].

We begin by evaluating a biologically inspired architecture that combines the hierarchical structure of deep convolutional networks with local recurrent dynamics within each visual area. CORnet was designed as a minimal, cortex-mappable model of the ventral stream, with just four areas — V1, V2, V4, and IT — each performing canonical neural computations such as convolution, normalization, and ReLU nonlinearities. This simplicity stands in contrast to modern, very deep CNNs, making CORnet particularly suitable for comparisons with anatomical and functional properties of visual cortex [14,31]. In CORnet, each area updates its state by integrating its current input with its own activity from the previous time step, according to:


$$\begin{array}{ccc} h_{t}^{\left(l\right)} & = & W(x_{t}^{\left(l-1\right)}+r_{t-1}^{\left(l\right)}) \end{array}$$ (1)

$$\begin{array}{ccc} r_{t}^{\left(l\right)} & = & \sigma (h_{t}^{\left(l\right)}) \end{array}$$ (2)

where the input $x_{t}^{\left(l-1\right)}$ to area *l* is the current video frame when *l* is the first area (V1), and the output of the preceding area, $x_{t}^{\left(l-1\right)}=r_{t}^{\left(l-1\right)}$, for the downstream areas (V2, V4, IT). Here $r_{t-1}^{\left(l\right)}$ is the recurrent activation from the same area at the previous time step. Each area app*l*ies two convo*l*utions: a first convolution *W*_1_ that filters only the feedforward input $x_{t}^{\left(l-1\right)}$, and a second convolution *W* that integrates the resulting feedforward drive together with the recurrent activation $r_{t-1}^{\left(l\right)}$ within the same area (Figs 2 and S5). For notational simplicity, the first convolution *W*_1_ is omitted from the equations: the symbol $x_{t}^{\left(l-1\right)}$ already denotes the input after this first convolution, so that *W* in the update equations refers to the second convolution only. This recurrent update allows each layer to integrate information over time, a key feature missing in purely feedforward CNNs.

However, in this configuration, the model is entirely deterministic: its output is fully determined by the stimulus sequence. This makes it unsuitable for measuring intrinsic timescales, which require observing spontaneous fluctuations in neural activity across repeated presentations of the same input. As argued in [17], intrinsic timescales reflect the duration over which neural activity remains temporally correlated in the absence of stimulus variability—typically revealed only when internal variability (e.g., noise) is present. Therefore, while CORnet is a biologically plausible structure for exploring stimulus-driven dynamics and hierarchical response timescales, it lacks the internal variability necessary to investigate intrinsic dynamics. This motivates the introduction of stochastic noise as an additional component. We do so by adopting the following state update rule:


$$\begin{array}{ccc} h_{t}^{\left(l\right)} & = & W(x_{t}^{\left(l-1\right)}+r_{t-1}^{\left(l\right)})\;+\eta_{t}^{l} \end{array}$$ (3)

$$\begin{array}{ccc} r_{t}^{\left(l\right)} & = & \sigma (h_{t}^{\left(l\right)}) \end{array}$$ (4)

where $\eta_{t}^{\left(l\right)}\sim 𝒩(0,\sigma^{2}$) represents additive Gaussian noise injected into each layer to reproduce biological variability across trials (see Methods for details on the scale of the noise).

We then tested whether this minimal model is capable of reproducing the experimentally observed hierarchy of response and intrinsic timescales. As mentioned above, this setup corresponds to a widely held hypothesis in computational neuroscience: that spatial invariance alone can lead to temporal stability. Under naturalistic stimulation, neurons in higher-order visual areas—such as IT—are thought to maintain more stable responses over time due to their invariance to object transformations, while early visual areas like V1 respond more transiently to low-level visual features rapidly changing in the input. Accordingly, one might expect that the progressive increase in spatial invariance along the ventral stream would be mirrored by a corresponding increase in temporal stability. CNNs, with their hierarchical architecture and increasing receptive field size, are designed to implement precisely such a progression.

**Fig 3:**
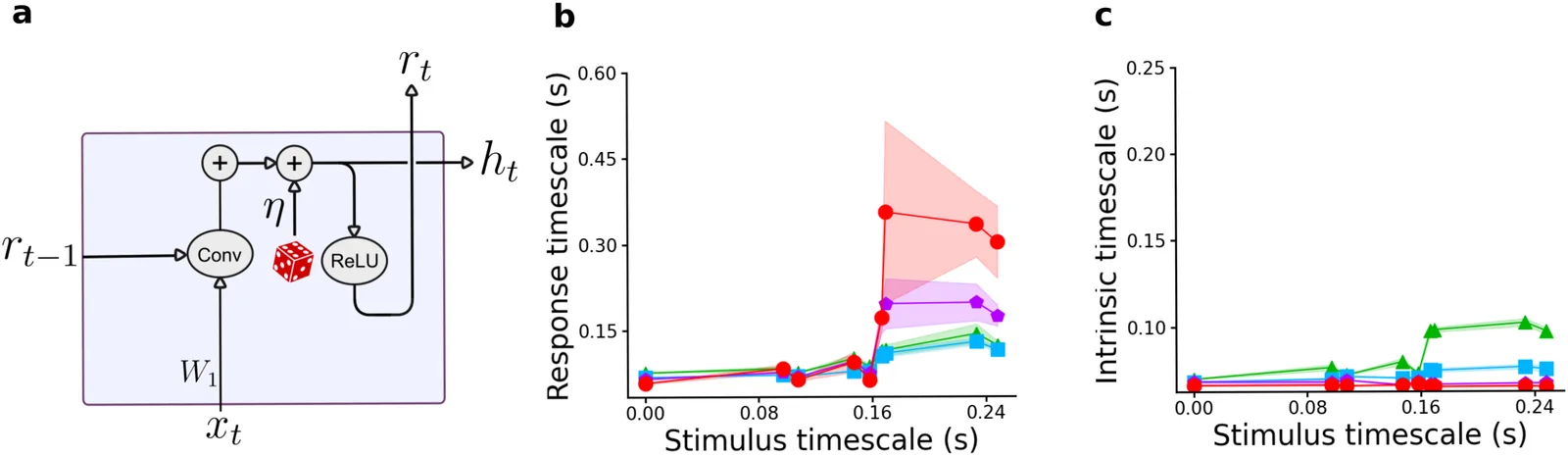
Response and intrinsic timescales in Stochastic CORnet model. **a)** Circuitry within an area of the Stochastic CORnet model, corresponding to the equations (Eq. 4). Here, $h_{t}$ is the pre-activation variable, $r_{t}=\sigma (h_{t})$ is the post-activation unit activity (ReLU output), and $\eta_{t}$ the additive Gaussian noise. *W*_1_ denotes the convolution that filters only the feedforward input $x_{t}$ (from the visual stimulus or the preceding area), whereas the **Conv** block represents the second convolution integrating both the feedforward and the recurrent signals within the same area; **b)** Response timescales; **c)** Intrinsic timescales. For each area, the timescale was computed by averaging the estimates over 5 different subsets of 200 randomly selected units from the corresponding layer. Shaded areas represent the standard deviation across subsets (see Methods).

However, our results show that spatial invariance alone is not sufficient to produce a biologically realistic hierarchy of timescales. Although response timescales show a weak increasing trend across areas, this pattern is stepwise rather than smoothly graded, and much less consistent than in the experimental data (Fig 3). More strikingly, the intrinsic timescales show the opposite trend: they decrease along the hierarchy (Fig 3). In other words, activity in early visual areas like V1 remains temporally correlated over longer periods than in downstream regions, failing to reproduce the experimental findings from electrophysiological recordings.

### Leaky CORnet

Increasing temporal stability can also arise from mechanisms beyond spatial invariance alone. A prominent alternative is the intrinsic dynamics of the network: neurons may exhibit a form of temporal inertia, whereby their current activity reflects a memory of recent past states, even when external inputs vary rapidly. This is in line by previous work suggesting that intrinsic timescales can emerge from autoregressive dynamics, where each state is shaped by its own temporal history [17,18]. To test this possibility, we introduced a leak term in the network dynamics, controlling the auto-regressive character (or “inertia”) of neural activity. Specifically, for each area, the dynamics evolve according to:


$$\begin{array}{ccc} h_{t}^{\left(l\right)} & = & W(x_{t}^{\left(l-1\right)}+r_{t-1}^{\left(l\right)})+(1-\gamma )\;h_{t-1}^{\left(l\right)}+\eta_{t}^{\left(l\right)} \end{array}$$ (5)

$$\begin{array}{ccc} r_{t}^{\left(l\right)} & = & \sigma (h_{t}^{\left(l\right)}) \end{array}$$ (6)

where γ modulates the balance between input-driven updates and persistence of previous states. Larger values of γ promote smoother temporal evolution of neural activity, even under dynamic stimuli, consistent with the autoregressive processes underlying intrinsic timescales. To model the hierarchical organization of temporal processing observed in the cortex, we assigned different values of γ to each area, decreasing along the visual hierarchy from V1 to IT. Specifically, we set $\gamma_{\text{V1}}=0.6,\gamma_{\text{V2}}=0.5,\gamma_{\text{V4}}=0.4$, and $\gamma_{\text{IT}}=0.3$. Since the effective leak rate is given by 1−γ, areas with lower γ values retain more of their previous activity, resulting in a slower decay of autocorrelation over time.

**Fig 4:**
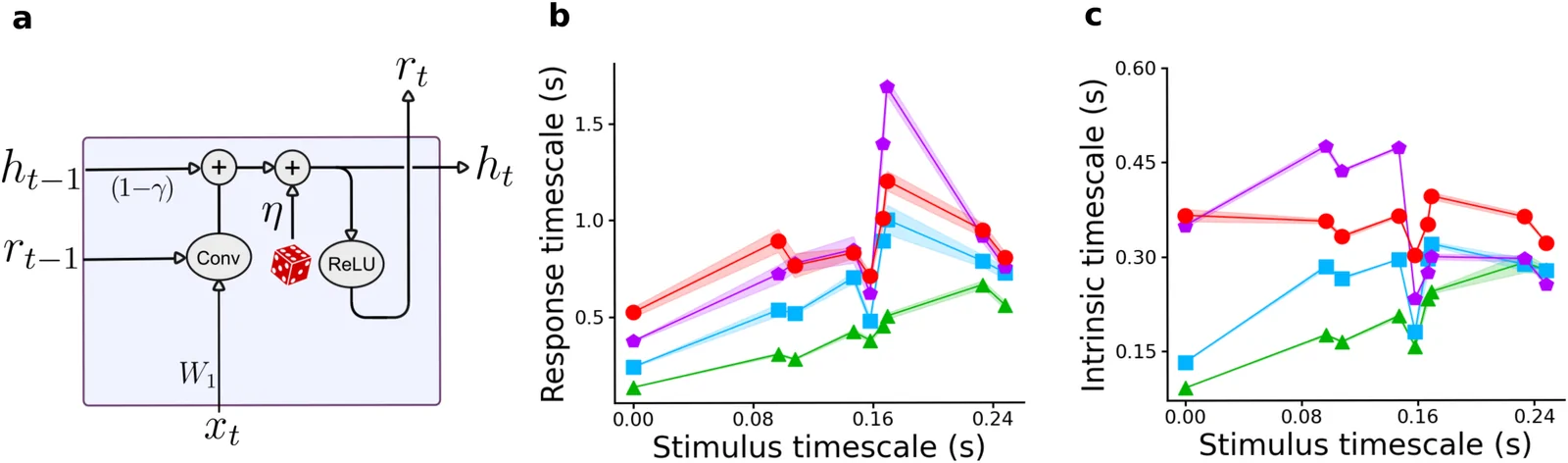
Response and intrinsic timescales in Leaky CORnet model. **a)** Circuitry within an area of the Leaky CORnet model, corresponding to the equations (Eqs. 5 and 6). Relative to the Stochastic CORnet, this variant includes an effective leak rate 1−γ, controlling the temporal integration of activity of the hidden state $h_{t}$ over time; **b)** Response timescales; **c)** Intrinsic timescales. For each area, the timescale was computed by averaging the estimates over 5 different subsets of 200 randomly selected units from the corresponding layer. Shaded areas represent the standard deviation across subsets (see Methods).

However, the combination of input recurrence and leaky integration does not yield a consistent hierarchy of timescales across areas. Despite the presence of both feedback from prior responses and temporal inertia, this configuration fails to produce the progressive increase in timescales observed experimentally (Fig 4).

### Adaptive CORnet

To further investigate the mechanisms that can shape intrinsic temporal dynamics beyond simple leaky integration, we introduced a model incorporating a form of neuronal adaptation. Specifically, we hypothesized that the current response of each neuron could be modulated by a memory trace of its past output, integrated over time with a tunable timescale. This idea is formalized through an auxiliary variable $s_{t}$, defined as a low-pass filtered version of the past neuronal responses and modulate the input drive by subtracting this adaptation signal:


$$\begin{array}{ccc} s_{t}^{\left(l\right)} & = & \alpha s_{t-1}^{\left(l\right)}+(1-\alpha )r_{t-1}^{\left(l\right)} \end{array}$$ (7)

$$\begin{array}{ccc} h_{t}^{\left(l\right)} & = & W(x_{t}^{\left(l-1\right)}-\beta s_{t}^{\left(l\right)})+(1-\gamma )h_{t-1}^{\left(l\right)}+\eta_{t}^{\left(l\right)} \end{array}$$ (8)

$$\begin{array}{ccc} r_{t}^{\left(l\right)} & = & \sigma (h_{t}^{\left(l\right)}) \end{array}$$ (9)

This formulation builds on the intrinsic suppression model by [39], augmenting it with noise and an autoregressive memory component. For each unit, α determines the integration window of the suppression mechanism. Larger α values correspond to longer memory and slower adaptation; smaller values emphasize recent activity. The other parameter, β, controls the strength of suppression. β>0 yields activity-dependent suppression; β<0 implements potentiation instead.

In analogy with the leaky model, the parameter γ was set independently for each area to reflect area-specific temporal integration. In contrast, α=0.96 and β=0.06 were kept constant across all layers [39]. To verify that these results do not depend critically on the specific choice of hyperparameters, we performed a sensitivity analysis in the (γ,α,β) parameter space (see S2, S3, S4 Figs).

**Fig 5:**
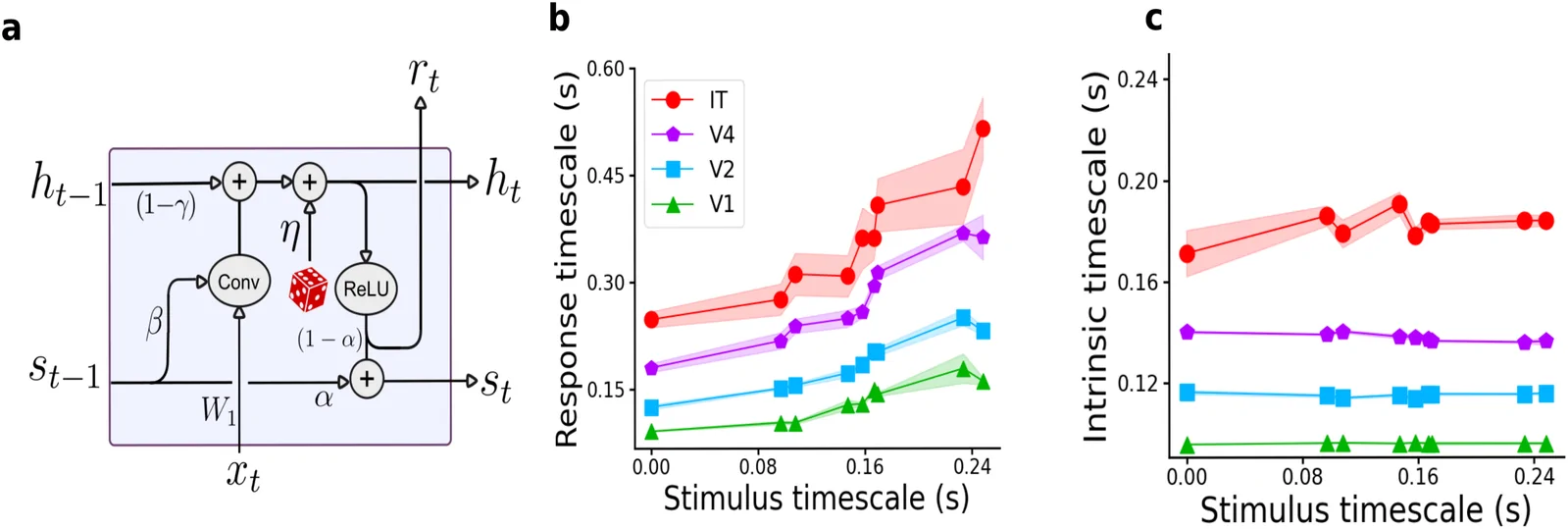
Response and intrinsic timescales in Adaptive CORnet model. **a)** Circuitry within an area of the Adaptive CORnet model, corresponding to Eqs 9. Relative to the Leaky CORnet, this variant introduces an adaptation variable $s_{t}$, updated as a low-pass filtered version of the past neuronal activity with timescale α, and subtracted from the input drive with strength β. These two parameters jointly determine the temporal profile of adaptation, shaping how past activity suppresses or enhances current responses. **b)** Response timescales; **c)** Intrinsic timescales. For each area, the timescale was computed by averaging across 5 subsets of 200 randomly selected units. Shaded regions indicate the standard deviation across subsets (see Methods).

We found that this model is able to reproduce the correct hierarchy of both response and intrinsic timescales (Fig 5), at the same time allowing interactions among neurons within the same layer. Such cross-recurrence is essential in CNN-RNN models aiming to mimic cortical circuitry, as it provides the basis for population-level dynamics and spatially distributed temporal integration.

### A minimal model of intrinsic dynamics

Interestingly, we find that even a minimal modification of CORnet, introducing a stochastic drive and a simple leaky integration term, can give rise to a realistic hierarchy of timescales. Specifically, we consider the following simplified dynamics:


$$\begin{array}{ccc} h_{t}^{\left(l\right)} & = & W(x_{t}^{\left(l-1\right)})+(1-\gamma )h_{t-1}^{\left(l\right)}+\eta_{t}^{\left(l\right)} \end{array}$$ (10)

$$\begin{array}{ccc} r_{t}^{\left(l\right)} & = & \sigma (h_{t}^{\left(l\right)}) \end{array}$$ (11)

**Fig 6:**
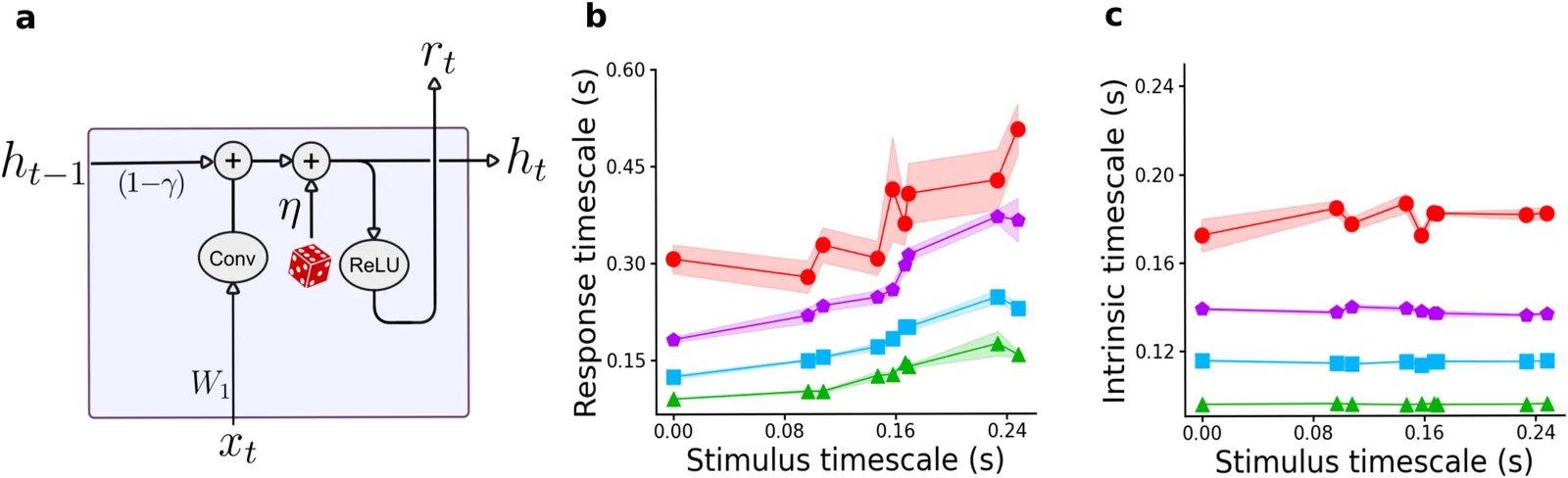
Response and intrinsic timescales in Minimal CORnet model. **a)** Diagram depicts circuitry within an area of Minimal CORnet, corresponding to Eq 11. Even without propagating the previous response $r_{t-1}$ through the convolution *W*_1_ - neither as in the original CORnet nor in the adaptation model — and by introducing only an effective leak term (1−γ), the network already exhibits a minimal form of intrinsic dynamics that reproduces the expected hierarchy of temporal processing across areas and relative to the stimulus timescale.; **b)** Response timescales; **c)** Intrinsic timescales. For each area, the timescale was computed by averaging the estimates over 5 different subsets of 200 randomly selected units from the corresponding layer. Shaded areas represent the standard deviation across subsets.

This formulation eliminates both recurrence from previous outputs and adaptation mechanisms, relying solely on the interaction between noisy inputs and leaky accumulation of past states. Despite its simplicity, this model captures a key feature of cortical dynamics: the progressive increase in temporal stability along the ventral stream. By assigning decreasing values of γ from early to late visual areas — thus increasing the effective memory of past activity — we obtain a monotonic hierarchy of intrinsic timescales that matches neurophysiological recordings (Fig 6).

### The role of learned computations

**Fig 7:**
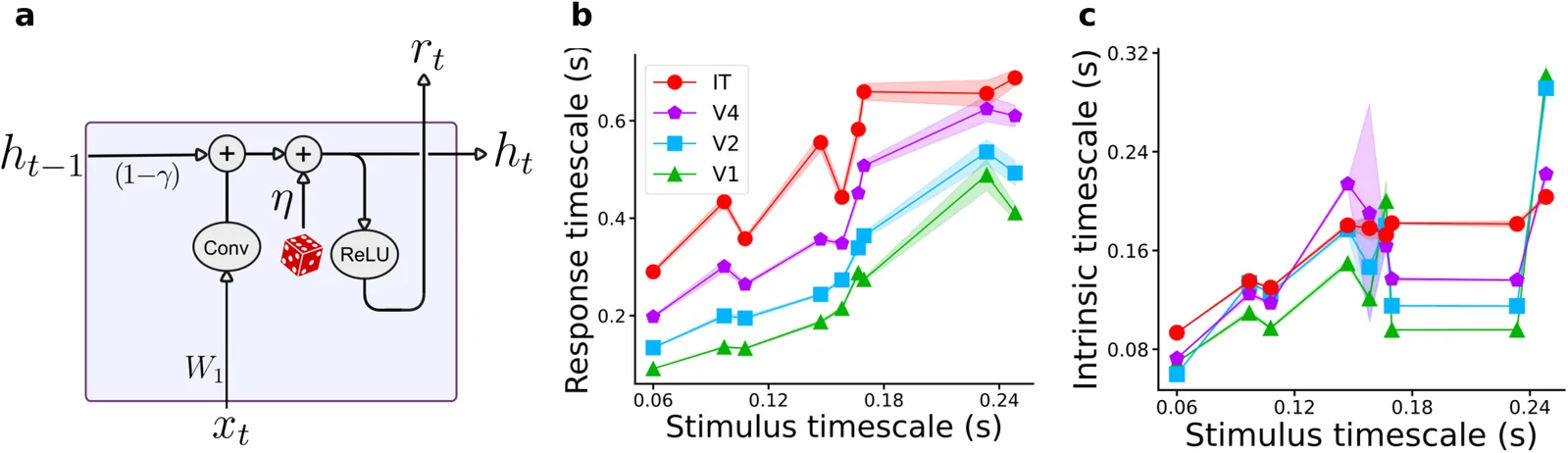
Response and intrinsic timescales in Random CORnet model. **a)** Diagram depicts circuitry within an area of Random CORnet. The architecture is identical to the Minimal CORnet model, except that all convolutional weights are randomly initialized rather than pretrained on ImageNet for object recognition; **b)** Response timescales; **c)** Intrinsic timescales. For each area, the timescale was computed by averaging the estimates over 5 different subsets of 200 randomly selected units from the corresponding layer. Shaded areas represent the standard deviation across subsets.

To assess whether the emergence of temporal hierarchies depends on the learned computations of the network, we introduced a control version of the model in which all weights are randomly initialized rather than pretrained on ImageNet. This variant preserves the same dynamical architecture of the minimal model, i.e., stochasticity and leaky integration, but removes any structured knowledge acquired through training. The motivation behind this manipulation is to disentangle the contributions of network architecture from those of learned representations. If the temporal hierarchy (i.e., increasing response timescales and intrinsic timescales across areas) were to persist in the absence of training, it would suggest that these properties emerge primarily from the network’s structural design. Conversely, if training is necessary for the correct hierarchy to appear, this would support the hypothesis that task-driven computations are essential for shaping the temporal dynamics observed in the ventral stream. Computational evidence suggests that although introducing a leak term and assigning area-specific γ values modulates the intrinsic persistence of neural activity, this mechanism alone is insufficient to generate a intrinsic temporal hierarchy. When the same leak dynamics are implemented in a network with randomly initialized weights — rather than using pretrained ImageNet weights — no systematic progression of intrinsic timescales across layers is observed (Fig 7). This indicates that the temporal hierarchy observed in trained networks emerges from an interaction between intrinsic temporal integration and task-driven learning.

We examine this interaction further in Supplementary Section *Spectral analyses of the convolutional weights and of the Jacobian of the dynamics*: a spectral analysis of the weights shows that the leading spatial eigenvalue does not increase along the hierarchy, and therefore cannot by itself explain the timescale ordering, while a Jacobian analysis indicates that learned weights instead shape the measured timescales through the gating of activity. Across model variants, the timescale hierarchy reflects the interaction between intrinsic integration and learned, stimulus-driven gating, rather than a systematic trend in the eigenvalues of the connectivity.

### Recurrent MLP: The effect of removing convolutional structure

To disentangle the effect of temporal recurrence $h_{t-1}$ from architectural constraints such as convolutional structure, we designed a minimal recurrent multilayer perceptron (MLP) that processes each frame of a video as a flattened visual input. Each layer integrates its past activity via a leaky update rule, without spatially structured operations such as convolutions. This allows us to investigate the role of temporal recurrence alone, in the absence of any spatial hierarchy. Each layer implements a dynamics:


$$\begin{array}{ccc} h_{t} & = & Wx_{t}+(1-\gamma )h_{t-1}+\eta_{t} \end{array}$$ (12)

$$\begin{array}{ccc} r_{t} & = & \phi (h_{t}) \end{array}$$ (13)

The matrix W represents a fixed random linear transformation mapping the input $x_{t}$ to the hidden units. It is initialized once and kept constant. The network comprises four layers with fixed width (Table 2). The MLP was (approximately) matched in terms of total number of parameters with CORnet-R; we note that in order to enforce this matching the input resolution for the MLP had to be significantly decreased (see Methods).

**Table 2:** Architecture of the recurrent MLP model. Each layer implements leaky temporal integration with no spatial structure and processes the output of the previous area through convolutional and recurrent computations, maintaining constant dimensionality from V2 onward.

Layer	Input Dimension	Number of Units
V1	3072	1024
V2	1024	1024
V4	1024	1024
IT	1024	1024

Despite the lack of convolutional structure, the model still exhibits temporal dynamics via the diagonal recurrent connections within each layer, enabling a comparison with more complex CNN-RNN architectures.

**Fig 8:**
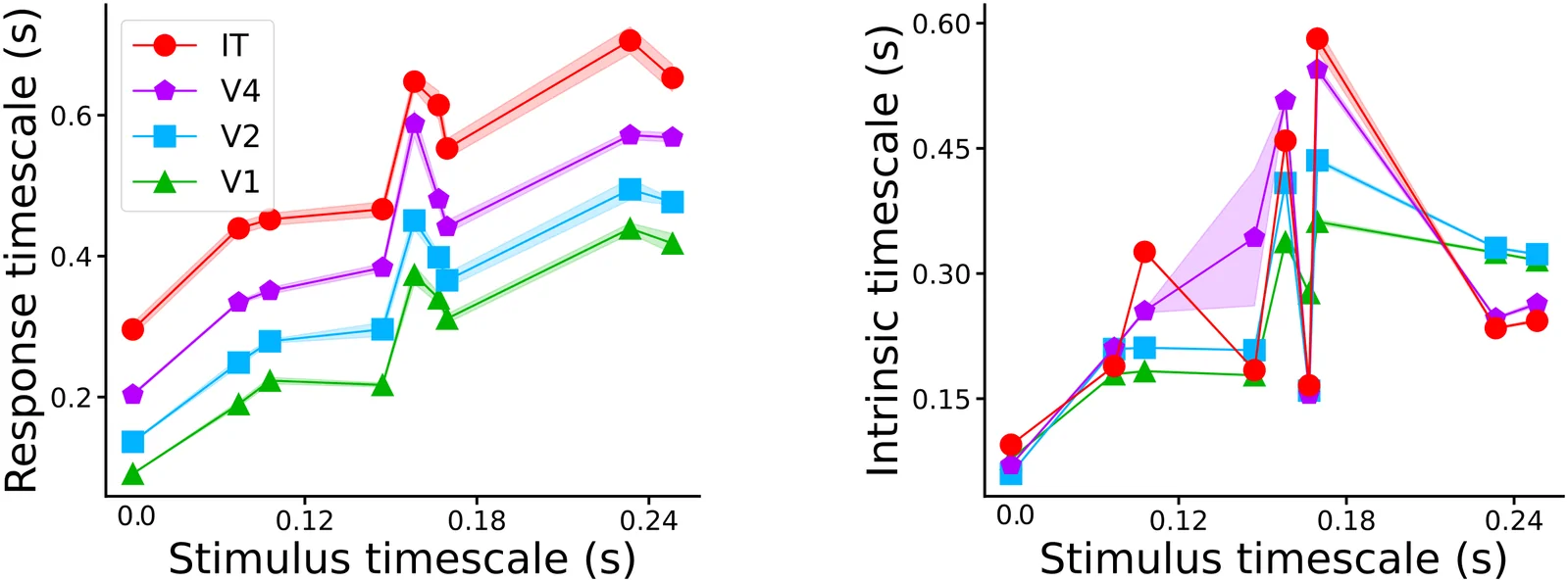
Response and intrinsic timescales in Randomly initialized chain of MLP. **a)** Response timescales; **b)** Intrinsic timescales. For each area, the timescale was computed by averaging the estimates over 5 different subsets of 200 randomly selected units from the corresponding layer. Shaded areas represent the standard deviation across subsets. Notably, intrinsic and response timescales exhibit lower variability compared to non-random cases, with average standard deviation across all points of $\sim 10^{-3}$.

We compared the response and intrinsic timescales across layers of the recurrent MLP model with decreasing leak rate along the hierarchy to those of CORnet with random weights. As shown in Fig 8, deeper layers exhibit slower responses to dynamic inputs. This result highlights that diagonal recurrence alone — without any convolutional structure or learned weights — can produce a hierarchical organization of response timescales, that is, progressively slower stimulus-evoked activity across layers. However, this effect does not extend to intrinsic timescales which do not form a consistent hierarchy, mirroring what is observed in CORnet with random weights. These findings suggest that while response timescales can arise from simple architectural gradients such as varying leak rates, the emergence of intrinsic timescale hierarchies in network models may require more complex mechanisms, such as structured recurrent connectivity or learning-induced dynamics.

### ImageNet classification performance

To evaluate the image recognition capability of our modified CORnet-RT network in its cross-adaptation configuration, we adopted a standard linear probing protocol [40,41]. We first extracted representations from the output layer of the IT block. For each image in the training and validation sets of ImageNet-1K, the model was run in evaluation mode for T = 5 recurrent time steps. This choice ensures consistency with the original CORnet training procedure. We retained the output of the final time step from IT, then applied average pooling followed by flattening to obtain a feature vector. These representations were then used to train a linear decoder. Feature extraction was performed in batches of 256 images. To estimate the variability of linear decoding performance, we trained the linear classifier multiple times (N = 3) with different random seeds, while keeping the extracted representations fixed. This approach, commonly used to assess representational quality, allows us to isolate the contribution of the learned features independently of the classifier training, and to test whether the internal modifications introduced in our model architecture preserve linearly decodable object representations. Despite no end-to-end fine-tuning, the minimal model yields a top-1 accuracy of 37.28% and a top-5 accuracy of 62%, indicating that the internal recurrent dynamics preserve linearly decodable high-level object representations. We note this result outperforms the accuracy reported for the recent CordsNet-R4 model (33.78%) [12], despite CordsNet undergoing an elaborate three-stage initialization and fine-tuning procedure specifically designed to optimize performance in continuous-time RNNs. The fact that it achieves competitive performance with significantly simpler training highlights the computational efficiency and architectural robustness of our design.

Compared to the original CORnet-RT model, our networks generally achieve a lower classification scores (on ImageNet-1K, CORnet-RT achieves 55.4% top-1 and 78.9% top-5 accuracy). However, we stress again that CORnet-RT is trained end-to-end for image classification. On the other hand, our models contain additional parameters and architectural modifications that are motivated by our biological question, but have not been optimized for task performance, as this is out of scope of the present work and its focus on representational dynamics. In this light, the performance difference with respect to CORnet-RT is a conservative upper bound to what would be obtained with a fairer comparison involving end-to-end training of our own networks. Below, we report the classification performance of all the model variants introduced to explore the hierarchy of timescales (Table 3). The accuracy progression of the linear decoder across training epochs is reported in S1 Fig.

**Table 3:** Top-1 and Top-5 classification accuracy for all model variants on the ImageNet-1K validation set. Accuracy values are reported as mean ± standard deviation across multiple validation runs.

Model	Top-1 Accuracy (%)	Top-5 Accuracy (%)
CORnet-RT	55.40 ± 0.08	78.90 ± 0.03
Stochastic CORnet-RT	54.23 ± 0.07	78.17 ± 0.03
Leaky CORnet-RT	32.38 ± 0.04	56.30 ± 0.04
Adaptive CORnet-RT	37.35 ± 0.04	61.60 ± 0.02
Minimal CORnet-RT	37.28 ± 0.03	61.55 ± 0.02
Random CORnet-RT	2.35 ± 0.47	6.75 ± 1.13

## Discussion

Our results demonstrate that an ordered, hierarchical progression of temporal dynamics in convolutional recurrent networks critically depends on the interaction between architectural features and dynamical mechanisms. In particular, while response timescales increase along the hierarchy even in untrained networks, intrinsic timescales only exhibit the experimentally observed progression when explicit memory mechanisms (such as leaky integration) and learned representations are in place. First, we showed that spatial invariance alone, as implemented in the Noisy CORnet-R model, is not sufficient to account for the hierarchy of intrinsic timescales observed in cortical recordings. This directly challenges the hypothesis that temporal stability of responses necessarily follows from increasing spatial pooling and feedforward depth. Second, introducing leaky integration in the Leaky-CORnet model successfully recovered the intrinsic timescale hierarchy, confirming that slow internal dynamics — controlled here by area-specific leak parameters — are sufficient to produce long-lasting activity fluctuations. This result aligns with prior experimental findings linking intrinsic timescales to local autoregressive dynamics in cortical circuits [17,18]. Third, adding cross-recurrence through adaptation mechanisms did not disrupt the hierarchy, as long as intrinsic memory was retained. Interestingly, we found that models in which the adaptation term dominated (i.e., with strong β) were prone to pathological autocorrelation profiles, limiting the biological plausibility of pure adaptation-based accounts. Instead, the combination of leaky dynamics and moderate adaptation yielded robust temporal hierarchies, supporting the idea that multiple recurrent mechanisms may coexist in shaping cortical dynamics. Finally, we found that training plays an essential role: in randomly initialized networks, the intrinsic hierarchy collapsed even when the architectural and dynamical features were preserved. This indicates that learned feature selectivity interacts nontrivially with internal dynamics, supporting the idea that intrinsic timescales are shaped by both structure and function. This complements and extends previous observations made in trained RNNs for action recognition tasks [42] and parallels findings in spiking neural networks trained on temporal tasks, where a hierarchy of neuronal time constants emerges spontaneously when these are optimized by gradient descent from homogeneous initial values [43]. Taken together, these findings point to a multifactorial origin of temporal hierarchies in visual cortex, involving both structural motifs (such as hierarchical depth and local recurrence) and functional constraints (such as task-driven learning and internal memory). In our view, evaluating models of cortical computation should go beyond static object recognition and include a dynamical characterization of both evoked and intrinsic timescales.

Our results connect to a substantial body of theoretical work on the origin of cortical timescales. A first line of work derives long population timescales from recurrent dynamics operating near a critical point, where the leading eigenvalue of an effective connectivity matrix approaches instability [44,45]. This recurrent route, however, is a fragile generative mechanism for a graded hierarchy: the divergence of the network timescale is a critical-slowing-down phenomenon confined to the immediate vicinity of the transition; the slowly decaying fluctuations carry vanishingly small amplitude precisely where their timescale diverges; and the divergence is suppressed by time-varying or stochastic input, which regularizes it into a finite maximum [46]. In our models the hierarchy does not rely on proximity to criticality: it is set by area-specific integration together with learned, stimulus-driven gating, and is therefore robust across a broad parameter range and under noise. A second, complementary line of work shows that biologically grounded gradients in recurrent circuits suffice to reproduce the hierarchy of intrinsic timescales without task training. Large-scale models of macaque cortex with anatomically derived connectivity and a hierarchical gradient of local recurrent strength reproduce the observed ordering of timescales [47], and related models capture their task-dependent modulation [48,49]. This is where our framework fills a gap. Existing dynamical accounts are typically not image-computable, while convolutional models of the ventral stream are evaluated almost exclusively on static recognition and lack a dynamical characterization [1].

A key limitation of our current approach is that the model is not trained end-to-end on an object recognition task. Instead, it relies on fixed pretrained weights obtained from CORnet-RT trained on ImageNet. While this allows us to isolate the effect of architectural and dynamical modifications on temporal processing, it likely underestimates the full representational capacity of the model. In particular, we expect that retraining the modified architecture end-to-end could improve classification performance, potentially matching or exceeding the accuracy levels of the original CORnet. Future work should explore whether such end-to-end training affects the emergence and structure of temporal hierarchies, and whether performance gains align with changes in intrinsic or response timescales. CORnet was originally designed as a model of the primate ventral visual cortex, whereas our comparisons are based on experimental data recorded from the rat visual system. Nevertheless, the dynamical principles examined here — such as leaky integration, adaptation, and recurrence — are likely conserved across species, reflecting general strategies for balancing temporal integration and responsiveness [47,50–53].

Our framework opens several directions for future work. One important extension would be to investigate the role of feedback connections in shaping temporal hierarchies. In biological circuits, feedback interacts continuously with sensory input, modulating integration and response timescales across areas. Incorporating feedback pathways that operate on comparable timescales to the feedforward drive could therefore reveal new forms of dynamic across the cortical hierarchy. Another promising direction would be to decouple the intrinsic dynamical timescale of the model from the frame rate of the visual input. In our current implementation, the temporal update of neural activity coincides with the presentation of consecutive video frames, effectively constraining the system’s dynamics to the timescale of the stimulus. Allowing the network to evolve on a finer temporal resolution — or to integrate multiple internal steps per frame — could uncover richer temporal behaviors, including oscillatory or predictive regimes that may otherwise be suppressed by forcing the dynamics to unfold in lockstep with the movie frames.

## Methods

### Network architecture

CNN models operate in a purely feedforward manner, lacking the rich lateral connections that are known to characterize the visual flow of the ventral region, and the complex response dynamics they produce. While classical CNNs trained for image recognition have achieved remarkable success, they remain incomplete models of the visual system, as they lack mechanisms for temporal integration. To address this limitation, the CORnet family of models [14] has been proposed as a more biologically inspired architecture for visual processing. In particular, CORnet-RT introduces local recurrent dynamics within each visual area (V1, V2, V4, IT), while maintaining a hierarchical feedforward structure. Each area performs biologically plausible computations, including convolutions, normalization, ReLU nonlinearities, and pooling (see Table 4 for details). The architecture is unrolled in time in a biologically realistic manner: At time *t* = 0, only the first layer is active; the second layer receives input from the first one at *t* = 1, etc, resulting in an input reaching the last layer only after four steps. Unlike standard machine learning implementations of recurrent models, which propagate input through all layers simultaneously at each time step, this stepwise temporal unrolling mimics the biologically plausible flow of information across areas (Figs 2 and S5). As such, it provides a useful framework for investigating hierarchical neural dynamics.

**Table 4:** Architectural parameters of the CORnet model. Each stage corresponds to one cortical area (V1, V2, V4, IT). The decoder stage performs average pooling, flattening, and a final linear layer producing 1000-class outputs.

Stage	Operation	Output shape
Input	–	224×224×3
V1	conv 7×7/4	56×56×64
	conv 3×3	56×56×64
V2	conv 3×3/2	28×28×128
	conv 3×3	28×28×128
V4	conv 3×3/2	14×14×256
	conv 3×3	14×14×256
IT	conv 3×3/2	7×7×512
	conv 3×3	7×7×512
Decoder	avgpool	1×1×512
	flatten	512
	linear	1000

The standard CORnet model ends with a linear decoder trained on ImageNet. At each time step, activity from the final stage is flattened and passed to a classifier, enabling object recognition across 1000 categories via a softmax layer. In the original CORnet-RT model, each visual area evolves over a fixed number of discrete time steps (typically times = 5), but the input image remains static throughout: at *t* = 0, the image is presented to V1, which updates its internal state; at *t* = 1, V1 passes its output to V2, and so on. This leads to temporal dynamics across areas, but they are not driven by a time-varying stimulus. To model neural responses to dynamic input, we modified CORnet-RT to process a sequence of video frames, feeding a new frame to V1 at each time step. Unlike the original implementation - which retains only the final output - we also store the full time sequence of activations across all areas. This enables detailed analysis of the evolving internal dynamics and allows us to compare model predictions to neural recordings obtained with movie stimuli.

To mimic biological trial-to-trial variability, we added, at each time step, Gaussian noise $\eta_{t}^{\left(l\right)}\sim 𝒩(0,\sigma^{2})$ to the hidden (pre-activation) state $h_{t}^{\left(l\right)}$ of each area, after the second convolution and normalization and together with the leak term (see Eq. 11 and S5 Fig); the noise thus enters the recurrent dynamics rather than being injected into the convolutional input. The same noise standard deviation σ was used for all areas. We selected the smallest value of σ that preserved the model’s functional performance while introducing sufficient trial-to-trial variability to estimate intrinsic timescales. As shown in Table 5, $\sigma =7\times 10^{-6}$ leads to only a minimal drop in classification accuracy relative to the original CORnet-RT, maintaining a top-1 accuracy of 54.23% and a top-5 accuracy of 78.17% on ImageNet-1K, compared to 55.4% and 78.9% respectively. Higher noise values severely compromise classification performance.

**Table 5:** Top-5 accuracy of the Stochastic CORnet-R model as a function of the noise standard deviation σ. Increasing the noise standard deviation σ progressively degrades the model’s classification performance, with accuracy dropping sharply beyond $\sigma =1.4\times 10^{-4}$.

Noise scale σ	Top-5 Accuracy (%)
$7\times 10^{-5}$	78.45
$1.4\times 10^{-4}$	74.52
$2.1\times 10^{-4}$	41.89
$2.8\times 10^{-4}$	10.40

All model variants share the same architectural backbone, temporal unfolding mechanism, and stochastic drive described above. To explore the role of different neural mechanisms in shaping intrinsic dynamics, we manipulated the recurrent update equations by including additional components—namely, leaky integration and adaptation. These components were instantiated by tuning a small set of hyperparameters (γ,α,β), whose values were kept fixed across trials and stimuli. Table 6 summarizes the parameter values used in each variant.

**Table 6:** Model parameters for different CORnet variants. The values of γ control leaky integration in each area, while α and β define the adaptation strength and memory. Dashes indicate that the corresponding mechanism was not used. Each CORnet-R variant uses distinct dynamical parameters.

Model variant	$\gamma_{V1}$	$\gamma_{V2}$	$\gamma_{V4}$	$\gamma_{IT}$	α	β
Leaky CORnet-R	0.6	0.5	0.4	0.3	–	–
Adaptive CORnet-R	0.6	0.5	0.4	0.3	0.96	0.06
Minimal CORnet-R	0.6	0.5	0.4	0.3	–	–

### Stimuli and network presentation protocol

The main stimulus set comprised nine video clips, each lasting 20 seconds and sampled at 30 frames per second (fps). The stimuli are described in depth in [19]. Briefly, six of the videos were naturalistic movies depicting real-world dynamic scenes, while the remaining three were synthetic controls: phase-scrambled versions of two natural movies and a white noise movie. The phase-scrambled movies were obtained by performing a spatiotemporal fast Fourier transform (FFT) over the standardized 3D array of pixel values, obtained by stacking the consecutive frames of a movie, and by shuffling the phases of the tranform (see [19] for details). The white-noise movie was generated by randomly setting each pixel in each frame either to white or black. Each video was composed by a sequence of 600 frames (20s × 30 fps), and each frame was input to the models we analyzed at a single time step *t*, such that the recurrence in *t*he network (i.e., the update of internal states $h_{t}$) coincided with the temporal evolution of the movie. In this formulation, each change in frame corresponds to a single recurrence of the internal dynamics governed by the update rule:


$$\begin{array}{c} h_{t}=W(x_{t}-\beta s_{t})+(1-\gamma )h_{t-1}+\eta \end{array}$$ (14)

Therefore, the framerate of the video stimuli provides the bridge between the networks’ discrete time and the time of neural activity, measured in seconds. This, in turn, results in a step duration of 33ms, which matches the bin size used for the analyses of the spiking data in [19].

### Estimation of temporal correlation

To quantify the temporal structure of both stimulus and neural signals, we computed the characteristic timescale via the lagged Pearson correlation function:


$$\begin{array}{c} C(\tau )=\frac{1}{T-\tau }\sum_{t=1}^{T-\tau }\frac{Cov[X_{t},X_{t+\tau }]}{\sqrt{Var[X_{t}]\;Var[X_{t+\tau }]}} \end{array}$$ (15)

This metric captures how quickly a time series decorrelates over time and was applied uniformly across three domains of analysis:

1. **Movie stimuli**: $X_{t}$ represents the intensity of the pixels in the movie frame at time *t*, and the (co)variances are estimated across pixels seen as independen*t* realization of the random variable $X_{t}$. For each lag τ, we computed the average correlation coefficient between all frame pairs separated by τ, yielding a global measure of how rapidly the visual content changed over time. This allowed us to assign a characteristic timescale to each movie.
2. **Stimulus-driven neural activity**: $X_{t}$ is the trial-averaged and temporally centered spike count at time *t*, i.e., $X_{t}=\{x_{n}(t)-\langle x_{n}\rangle_{t}\}$, and the (co)variances are estimated across neurons. This approach mirrors *t*he one used for the movies and characterizes the decay of evoked population activity across time lags.
3. **Intrinsic neuronal activity**: For a given neuron, $X_{t}$ is the activity at time bin *t*, and the (co)variances are estimated across *t*rials. The autocorrelation C(τ) was computed for each unit and then averaged across all neurons within a given area before extracting a characteristic time constant, following [17] and [19].

This unified correlation framework enables direct comparisons of temporal scales across visual input and neural responses, both evoked and intrinsic.

To estimate the characteristic timescale from these autocorrelation functions, following [19] we fit the empirical curves using a damped oscillatory model:


$$\begin{array}{c} C(\tau )=a\;e^{-\tau /\tau_{\text{fit}}}\cos(2\pi \omega \tau +\phi )+b \end{array}$$ (16)

Here, *a* is the amplitude, *b* the long-term offset, $\tau_{\text{fit}}$ the decay timescale, ω the oscillation frequency, and ϕ a phase shift. Parameters $\theta =(a,b,\tau_{\text{fit}},\omega ,\phi )$ were optimized by minimizing the squared error:


$$\begin{array}{c} ℒ(\theta )=\frac{1}{2}{\left\|C_{\text{model}}(\tau ;\theta )-C_{\text{data}}(\tau )\right\|}^{2} \end{array}$$ (17)

To avoid sensitivity to local minima, we used the differential evolution algorithm implemented in SciPy, with conservative parameter bounds, a population size of 250, and up to 500 iterations.

**Fig 9:**
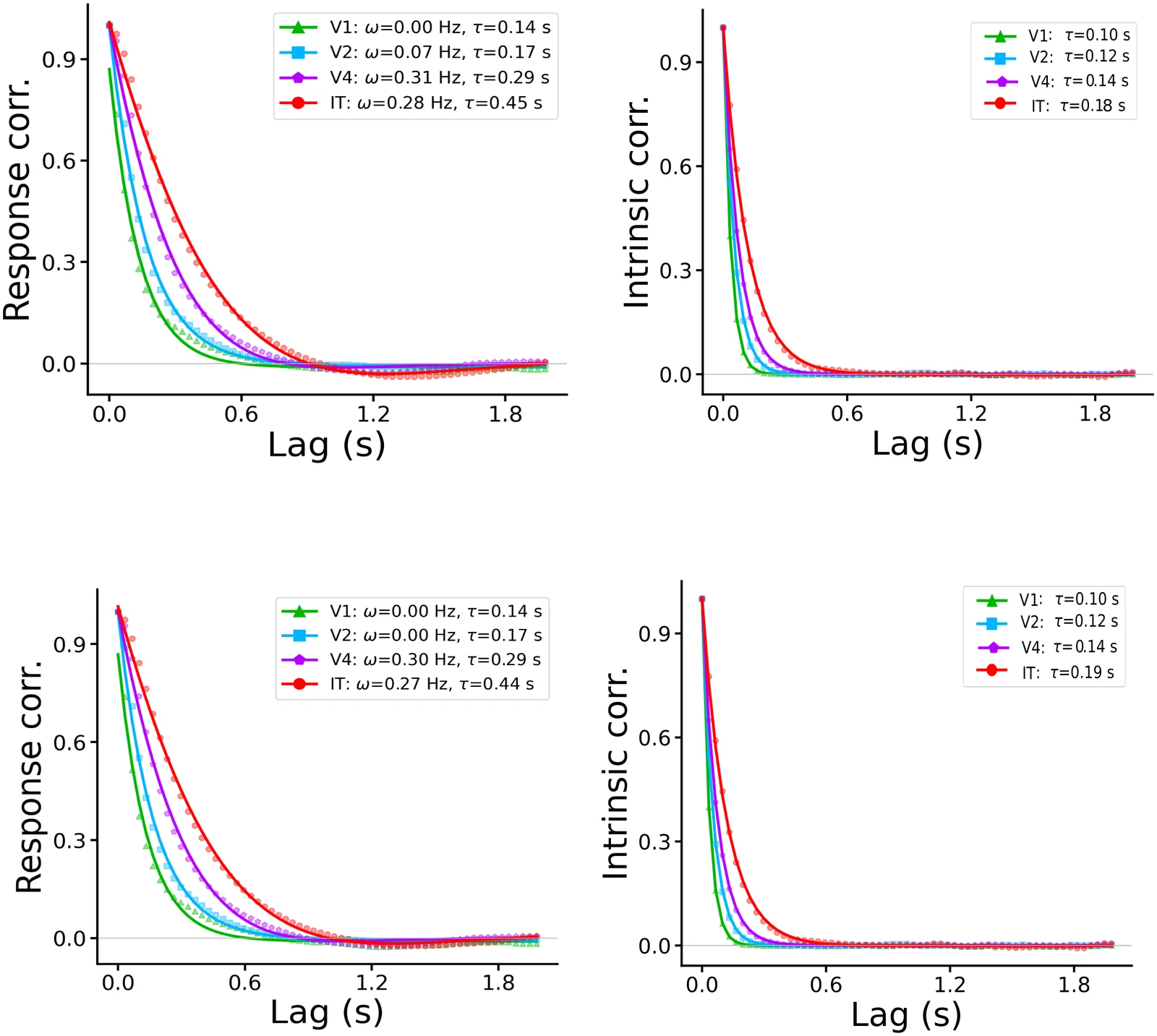
Autocorrelation functions and fitted timescales, response (left) vs. intrinsic (right), for the Minimal (top) and Adaptive (bottom) CORnet. Empirical ACFs (markers; mean across the five unit subsets) and fitted curves (solid lines) for the four areas (V1, V2, V4, IT), for a representative naturalistic movie (*manual slow 2*). The response ACFs are best fitted with the damped oscillatory model, while the intrinsic ACFs are well captured by a pure exponential decay. The parameters are $\gamma_{V1}=0.6$, $\gamma_{V2}=0.5$, $\gamma_{V4}=0.4$, $\gamma_{IT}=0.3$ in both models. For Adaptive CORNet, β=0.06 and α=0.96.

This fitting procedure was applied independently to each movie and to each cortical area (V1, V2, V4, IT). The fitted decay constant $\tau_{\text{fit}}$ served as a quantitative index of the timescale for both visual and neuronal autocorrelations. Model fits were visually inspected by overlaying the empirical and fitted curves (Fig 9).

### Design of the multilayer perceptron architecture

Our choice of parameters was guided by the need to match, as closely as possible, the total number of parameters of the CORnet-R architecture (approximately 4.7 million). To achieve this, we treated the parameter count as a design constraint: because each layer of the MLP performs a fully connected transformation rather than a convolutional one, every unit receives input from the entire visual field. As a result, maintaining the original input resolution would have led to an unfeasibly large number of parameters. We therefore reduced the input dimensionality to 3×32×32, which allows the overall parameter count to remain comparable to that of CORnet-R (around 6 million in our implementation) while preserving access to global spatial information in each layer.

### Evaluation of representational quality

To evaluate the representational quality of CORnet in its modified configurations, we employed a standard linear probing approach. All experiments were conducted in PyTorch using standard ImageNet preprocessing and evaluation protocols. Representations were extracted from the decoder output after five recurrent time steps, and a linear classifier was trained on frozen features. The full set of implementation parameters is reported in Table 7.

**Table 7:** Implementation details for the linear probing protocol used to evaluate CORnet-RT representations.

Component	Details
**Framework**	PyTorch 1.11.0
**Data**	ImageNet 2012 (Russakovsky et al., 2015)
**Preprocessing**	Resize to 256, center crop to 224×224 px, normalization using ImageNet mean and std
**Representations**	Extracted from CORnet-RT decoder (flatten layer) with 5 recurrent time steps
**Train set size**	1.28M images (ImageNet train split)
**Validation set**	50K images (ImageNet val split)
**Classifier**	Linear layer (input dim = 512, output dim = 1000)
**Training**	Features frozen; only the linear layer is trained
**Loss**	Cross-entropy
**Optimizer**	Adam
**Learning rate**	$1\times 10^{-3}$
**Batch size**	256
**Epochs**	1 (per run; repeated with different seeds)
**Metrics**	Top-1 and Top-5 accuracy (validation set)

## Supporting information

S1 Text Robustness to hyperparameter choices and spectral analyses. (PDF)

S1 Fig Linear decoding accuracy for pretrained CORnet-RT representations. (Left) Top-1 accuracy. (Right) Top-5 accuracy. A linear decoder was trained on frozen CORnet-RT features extracted from pretrained weights. Values represent the average over three independent training runs. Error bars (standard deviation) are scaled by a factor of 10 for improved visualization.
(TIFF)

S2 FigRobustness of the timescale hierarchy to alternative γ profiles.Response and intrinsic timescales of the Adaptive model under different area-specific leak parameter configurations. From top to bottom: (1) increased γ values across all areas, corresponding to uniformly lower leak rates (1−γ); (2) decreased γ values across all areas, resulting in uniformly higher leak rates; (3) constant γ values across areas, suppressing the hierarchical organization of timescales; (4) increasing γ values along the hierarchy, corresponding to an inverted leak-rate ordering. Left panels show response timescales as a function of stimulus timescale, while right panels show the corresponding intrinsic timescales. Each condition is compared with the baseline profile ($\gamma_{V1}=0.6,\;\gamma_{V2}=0.5,\;\gamma_{V4}=0.4,\;\gamma_{IT}=0.3$).(TIFF)

S3 FigRobustness of the timescale hierarchy to the adaptation parameters α and β in the Adaptive CORnet model.Response timescales (left column) and intrinsic timescales (right column) as a function of stimulus timescale, for the four areas, with the area-specific leak parameters held fixed at the baseline hierarchical profile ($\gamma_{V1}=0.6,\;\gamma_{V2}=0.5,\;\gamma_{V4}=0.4,\;\gamma_{IT}=0.3$). Each row corresponds to a different value of the suppression strength: (a) β=0.02, (b) β=0.04, (c) β=0.06. Within each panel, the three levels of marker opacity denote the adaptation timescale α∈{0.00,0.50,0.96}, from opaque to translucent. Across this range of α and β, both the ordering of the areas and the hierarchy of intrinsic timescales are preserved, with the curves for different α values nearly overlapping, indicating that the results are largely insensitive to the specific choice of adaptation parameters. Shaded areas represent the standard deviation across 5 subsets of 200 randomly selected units (see Methods).(TIFF)

S4 FigInterplay between adaptation strength β and the leak profile γ at the upper edge of the usable parameter range.(Top) Response autocorrelation function (ACF, left) and power spectral density (PSD, right) of each area under the white-noise stimulus, at β=0.08 with the *baseline* leak profile ($\gamma_{V1}=0.6,\;\gamma_{V2}=0.5,\;\gamma_{V4}=0.4,\;\gamma_{IT}=0.3$), for the same three α values (line style: solid α=0.0, dashed α=0.25, dash-dotted α=0.96). Whereas V1–V4 decay to zero within a few timesteps for all α, the IT ACF develops a long-lived component at the strongest adaptation (α=0.96), failing to return to baseline over the lag range shown; the PSD shows the corresponding low-frequency excess in IT. This marks the onset of a pathological regime at the baseline profile. In all panels, lines/markers denote the mean and shaded areas the standard deviation across 5 subsets of 200 randomly selected units (see Methods). (Bottom) Response (left) and intrinsic (right) timescales as a function of the stimulus timescale, by area (V1, V2, V4, IT), for the Adaptive model at β=0.08 with the leak profile ($\gamma_{V1}=0.8,\;\gamma_{V2}=0.7,\;\gamma_{V4}=0.6,\;\gamma_{IT}=0.3$). The three adaptation timescales α∈{0.00,0.25,0.96} are overlaid with increasing marker transparency. With this leak profile the four areas recover an ordered, well-separated hierarchy in both metrics ($\tau_{V1}<\tau_{V2}<\tau_{V4}<\tau_{IT}$), and the curves for different α nearly overlap, indicating that at this β the hierarchy is restored and remains largely insensitive to the adaptation timescale once γ is adjusted.(TIFF)

S5 FigUnrolled-in-time schematic of the Minimal CORnet-RT.Two areas (V1, green; V2, blue) are shown over three consecutive timesteps for the Minimal model. At each timestep a new movie frame enters V1, and activity propagates one area per timestep, so that V2 becomes active one step after V1, V4 one step after V2, and so on. The signal transmitted between areas is the post-nonlinearity activity r=σ(h). Specifically, the feedforward input received by area *l* at time *t* is the rectified output of area l−1 at the previous timestep, $x_{t}^{\left(l-1\right)}\equiv r_{t-1}^{\left(l-1\right)}$. (The subscript *t* labels the update step; the one-step propagation delay is carried by the fact that the input originates from the area below at t−1.) Within each area the only recurrence is the leaky integration of the hidden state, $(1-\gamma )h_{t-1}$; in the Minimal model the previous response $r_{t-1}$ is not fed back through the convolution. *W*_1_ denotes the convolution that filters the feedforward input, *W* the within-area convolution, and $\eta_{t}$ the per-area Gaussian noise. Each area carries its own leak constant $\gamma_{l}$, decreasing along the hierarchy. Video frame images adapted from [19].(TIFF)

S6 Fig Leading spatial eigenvalue of the convolutional weights across the hierarchy. *Solid blue* is for ImageNet-pretrained weights and *dashed orange* is for randomly initialized weights.
(TIFF)

S7 Fig Response and intrinsic timescales for the Adaptive model with randomly initialized weights. (Left) Response timescales. (Right) Intrinsic timescales, for the Adaptive model in the case of randomly initialized weights.
(TIFF)
