# Supplementary material for: Noisy models of the ventral stream reveal the impact of recurrence and learned representations on information processing timescales
Source: PLoS Comput Biol. 2026 Aug 27;22(8):e1014653. doi: 10.1371/journal.pcbi.1014653 (PMC13541256; doi:10.1371/journal.pcbi.1014653)
Supplement: Supplementary file 1 [file pcbi.1014653.s001.pdf]

S1 Text. Robustness to hyperparameter choices and spectral analyses

Robustness to hyperparameter choices

To assess whether the hierarchy of timescales depends on the specific numerical choice of the area-specific leak parameters, we tested the Adaptive model under several alternative γ profiles while keeping all other settings fixed, and compared each against our baseline profile ($\gamma_{V1} = 0.6$, $\gamma_{V2} = 0.5$, $\gamma_{V4} = 0.4$, $\gamma_{IT} = 0.3$). Specifically, we evaluated profiles in which all γ values were uniformly shifted upward, resulting in lower leak rates $(1 - \gamma)$ across all areas, as well as profiles with uniformly decreased γ values, corresponding to higher leak rates. Additionally, we tested a homogeneous γ profile with identical values across areas, and an increasing γ profile across the hierarchy, which effectively inverted the leak-rate ordering (S2 Figure). Overall, these controls show that qualitatively the timescale rankings do not depend on the specific choice of γ, although it is possible to invert the intrinsic timescale ordering by picking γ values that increase sufficiently rapidly along the anatomical hierarchy.

To further assess the robustness of our results, we systematically explored the effect of varying the α parameter across different values of β, while keeping the remaining model parameters fixed. This analysis allowed us to determine whether the observed timescale organization and model behavior were preserved across a broader range of parameter configurations (S3 Figure). We additionally examined cases with high β values, which introduced a slow component in the temporal dynamics of the signal (S4 Figure). In these regimes, the resulting response autocorrelation functions exhibited a prolonged decay, failing to return to baseline within the lag range relevant for comparison with experimental data. Such slow-decaying correlations are inconsistent with physiologically plausible temporal dynamics and therefore identify parameter regimes that do not capture the timescale structure observed in biological recordings. Crucially, the effect of β is not independent of the leak profile γ: the two parameters act jointly on the slow dynamical mode introduced by the adaptation. Increasing β strengthens this slow component, whose weight in the autocorrelation is set by the separation between the adaptation timescale and the intrinsic, leak-determined timescale of each area. With the baseline leak profile, this separation becomes too small in the deepest areas, so that at high β the IT autocorrelation develops a long-lived component and fails to return to baseline within the lag range relevant for comparison with experimental data. Re-tuning the leak profile (to $\gamma_{V1} = 0.8$, $\gamma_{V2} = 0.7$, $\gamma_{V4} = 0.6$, $\gamma_{IT} = 0.5$) widens this separation and restores monotonically decaying, non-pathological autocorrelations, together with the ordered timescale hierarchy.

Spectral analyses of the convolutional weights and of the Jacobian of the dynamics

To characterize how learned feature selectivity interacts nontrivially with the internal dynamics, we performed two complementary analyses: (i) the eigenspectra of the convolutional weight tensors and (ii) the Jacobian of the linearized dynamics. To assess whether the hierarchy of timescales could be attributed to a systematic trend in the spectral properties of the learned weights, we analyzed the spatial structure of the convolutional kernels of each area, separately for the ImageNet-pretrained and the randomly initialized condition. We followed the approach of Guth et al. (2024): each $k \times k$ kernel of an area's weight tensor $W^{(l)} \in \mathbb{R}^{C_{\text{out}} \times C_{\text{in}} \times k \times k}$ is treated as a sample in $\mathbb{R}^{k^2}$, and the $k^2 \times k^2$ spatial covariance is estimated by averaging over all $(c_{\text{out}}, c_{\text{in}})$ channel pairs and diagonalized to obtain its eigenvalues $\lambda_1 \geq \cdots \geq \lambda_{k^2}$. We find that the leading spatial eigenvalue λ_1 *decreases* along the hierarchy (V1→IT) in both conditions (S6 Figure) (at least in the randomized case, it is easy to explain this decrease as a direct consequence of the He weight initialization rule (He et al. 2015) (the PyTorch default), under which weights are drawn with a standard deviation inversely proportional to the square root of the fan-in; in CORnet the fan-in increases with layer depth, as in most convolutional neural networks).

The increase of timescales across areas cannot therefore be attributed to a simple trend in the spectral properties of the weights, as our observations contradict the standard intuition from recurrent neural networks, where larger recurrent leading eigenvalues yield slower dynamics. The observed timescale ranking must instead reflect a more complex interaction between learned feed-forward computations and recurrent processing. Finally, one must remember that despite the fact that the matrices $W^{(l)}$ are the same across all model variants, their role in network dynamics differs. We expand on this below.

Jacobian of the dynamics. In the Minimal model, $W^{(l)}$ does not enter the recurrent operator: the only term feeding the state back in time is the leak $(1-\gamma^{(l)})h_{t-1}^{(l)}$, while $W^{(l)}$ acts solely on the feedforward drive $x_t^{(l-1)}$. This does not mean that $W^{(l)}$ is irrelevant to the measured timescales — since we estimate them from $r_t^{(l)} = \sigma(h_t^{(l)})$, the learned weights still shape the autocorrelation through the gating of the rectified activity. The recurrent dynamics are

$$h_t^{(l)} = W^{(l)} x_t^{(l-1)} + (1-\gamma^{(l)}) h_{t-1}^{(l)} + \eta_t^{(l)}, \qquad r_t^{(l)} = \sigma(h_t^{(l)}), \tag{1}$$

so that the same-layer state-to-state Jacobian and the read-out Jacobian read

$$\frac{\partial h_t^{(l)}}{\partial h_{t-1}^{(l)}} = (1-\gamma^{(l)}) I, \qquad J_t^{(r \leftarrow h)} \equiv \frac{\partial r_t^{(l)}}{\partial h_{t-1}^{(l)}} = (1-\gamma^{(l)})\, D_t^{(l)}, \tag{2}$$

where $D_t^{(l)} = \mathrm{diag}(\sigma'(h_t^{(l)})) = \mathrm{diag}(\mathbf{1}_{h_t^{(l)}>0})$. The leak factor $(1-\gamma^{(l)})$ sets the linearized persistence of perturbations, while learning affects timescales through the statistics of the gating process, since $D_t^{(l)} = \mathrm{diag}\big(\mathbf{1}_{W^{(l)} x_t^{(l-1)} + (1-\gamma^{(l)}) h_{t-1}^{(l)} + \eta_t^{(l)} > 0}\big)$. Thus trained vs. random $W^{(l)}$ can strongly change which units remain active over time — and hence which fluctuations contribute to the measured intrinsic timescales — even when $\gamma^{(l)}$ is held fixed.

In the Adaptive model, by contrast, $W^{(l)}$ *does* enter the recurrent dynamics directly, through the adaptation feedback $-\beta W^{(l)} s_t^{(l)}$, since the trace $s_t^{(l)}$ integrates past activity $r_{t-1}^{(l)}$. Taking $(h^{(l)}, s^{(l)})$

as the state, the one-step Jacobian reads

$$\mathcal{J}_t^{(l)} = \begin{pmatrix} (1-\gamma^{(l)})\, I - \beta(1-\alpha)\, W^{(l)} D_{t-1}^{(l)} & -\alpha\beta\, W^{(l)} \\ (1-\alpha)\, D_{t-1}^{(l)} & \alpha\, I \end{pmatrix}, \qquad D_{t-1}^{(l)} = \mathrm{diag}\big(\mathbf{1}_{h_{t-1}^{(l)}>0}\big). \tag{3}$$

Here the weight matrix appears explicitly in the linearized recurrence, so that analyzing its spectrum becomes pertinent. We therefore replicated the randomization analysis for this variant (S7 Figure), finding that the timescale hierarchy is not preserved. Two features nonetheless distinguish it from a classical fixed-spectrum recurrent network. First, $W^{(l)}$ enters gated by the trajectory-dependent active set $D_{t-1}^{(l)}$ and scaled by the adaptation strength β, so its spectral radius does not control the dynamics on its own. Second, the block structure of $\mathcal{J}_t^{(l)}$ carries a slow eigenvalue $\approx \alpha$, associated with the adaptation trace, with timescale $\tau_s \approx 1/(1-\alpha)$ set by α and independent of $W^{(l)}$. The hierarchy thus reflects the joint action of the leak $\gamma^{(l)}$, the adaptation (α, β), and the gated weights, rather than the spectrum of $W^{(l)}$ alone (see also S2 Figure and S3 Figure).
