# Supplementary material for: Noisy models of the ventral stream reveal the impact of recurrence and learned representations on information processing timescales
Source: PLoS Comput Biol. 2026 Aug 27;22(8):e1014653. doi: 10.1371/journal.pcbi.1014653 (PMC13541256; doi:10.1371/journal.pcbi.1014653)
Supplement: Supplementary file 2 [file pcbi.1014653.s010.pdf]

**Response to Reviewers**

*Noisy models of the ventral stream reveal the impact of recurrence and learned representations on information processing timescales*

Dear Editor,

Thank you for your careful consideration of our manuscript. To address the points raised by the reviewers, we have performed additional analyses, added 4 new figures, revised existing figures, and rewritten, clarified and expanded several key passages of our text. For ease of comparison, in the submitted version of the text we have highlighted our changes in cyan. We believe that this version of the paper fully addresses the reviewers' concerns and is much clearer and stronger than our initial submission.

In the following, we respond to each point in more detail.

# Reviewer #1

> This manuscript studies the network mechanisms behind the hierarchical organization of temporal timescales in the visual ventral stream. Inspired by recent experimental results in rodent visual cortex, the authors try to reproduce the finding that both "response timescales" (the decay of trial-averaged activity) and "intrinsic timescales" (the decay of noise correlations) gradually increase along the anatomical hierarchy. The authors use several modified versions of CORnet-RT (Stochastic, Leaky, Adaptive, Minimal, and Random) to separately examine the roles of recurrence, spatial pooling, intrinsic cellular memory (leaky integration), and learning. Overall, these model variants are well-designed and well-suited for exploring different possible mechanisms. One important result is the clear separation between the two types of timescales. The authors show that response timescales and intrinsic timescales depend on different factors. Response timescales naturally arise from the deep convolutional structure, while intrinsic timescales do not. In addition, results from the stochastic CORnet suggest that spatial invariance and standard recurrence alone are not enough to produce a hierarchical organization of intrinsic timescales. Instead, explicit leaky integration or adaptation, together with heterogeneous time constants across layers, is required. Another key result concerns learning. Leaky integration by itself is not sufficient; it needs to be combined with learned representations. When the network weights are randomized, the hierarchy of intrinsic timescales disappears, even though the hierarchical leak constants are still present. Overall, this is a timely and interesting study that helps bridge the gap between static deep learning models of vision and the dynamic, temporally structured activity seen in biological neural circuits. The paper is suitable for acceptance, but there are still some concerns:

We thank the reviewer for the nice summary of our work and for appreciating the timeliness and potential impact of our study. We address each of the points below.

### R1.1

> The "Minimal Model" depends on manually setting the leaky parameter $\gamma$ to decrease monotonically. While the result that this alone is insufficient without learning is insightful, the claim that the hierarchy "emerges" in the Minimal Model may be somewhat overstated.

**Response.** Thanks. Yes, this partly overlaps with a question by R3 on the robustness of our findings vs changes in the hyperparameters of our models and one by R2 on the role of the leak. We have now performed additional analyses (supplementary Fig.2) that show that,

qualitatively, the observed trend in the minimal model does not depend on the specific values we used for the leak parameter $\gamma$, and is not fully abolished by removing the constraint that $\gamma$ decreases monotonically across network layers. Indeed, setting $\gamma$ to a constant throughout the network results in a strong compression of the differences in intrinsic timescales, but it still results in a more ordered progression of timescales than what we observe when randomizing the feedforward prediction weights (while still keeping $\gamma$ to our original values), Fig 7. On the other hand, it is possible to alter the hierarchy of the intrinsic timescales by setting the $\gamma$ values to be monotonically increasing across layers. Overall, we agree that it is important to point out more clearly that hyperparameter choices can make a difference, and we do so now in the results section, where we have toned down our language about "emergence".

## R1.2

> The novel finding "learned feature selectivity interacts nontrivially with internal dynamics" would be strengthened, by including a spectral analysis of the recurrent weight matrices or the Jacobian of the dynamics for the Trained and Random models".

**Response.** This is an insightful observation that also overlaps with some points raised by R2. In the simplest possible terms, the intuition here is that in an idealized, linear case for the dynamics along an eigenvector of a recurrent weight matrix associated with an eigenvalue $\lambda$, $h_t = x_t + \lambda h_{t-1}$ (assuming that $x_t$ contains both the time-varying input and the noise), so $h_t$ is simply the the discrete convolution between $x_t$ and an exponential kernel $\exp(-t/\tau)$, where $\tau = -1/\ln(\lambda)$. As long as $0 < \lambda < 1$, the dynamics are then expected to be slower as $\lambda$ approaches 1. Therefore, the reasoning goes, if the spectral properties of the recurrent weight matrix changed systematically along the model hierarchy — for instance with the largest eigenvalue being larger in deeper layers — this would provide an effective, mechanistic explanation for the observed hierarchy of timescales.

We performed spectral analyses of the synaptic weight matrices W for the pretrained and randomized condition, and we report the results in Supplementary Figure 5. As expected, the magnitude of the leading spatial eigenvalue $\lambda_1$ differs between the two conditions. More surprisingly, $\lambda_1$ *decreases*, rather than increasing, along the hierarchy (V1→IT) in both cases. Therefore the increase of timescales across areas cannot be attributed to a simple trend in the spectral properties of the weights, and must instead reflect a more complex interaction between the learned feedforward computations and the recurrent processing. The decrease of $\lambda_1$ with depth is, at least in the randomized case, a direct consequence of the He weight initialization rule (He et al. 2015) (the PyTorch default), under which weights are drawn with a standard deviation inversely proportional to the square root of the fan-in. In a convolutional architecture of constant kernel size such as CORnet, the fan-in grows with depth (deeper areas have more input features), so that individual weights are sampled from an increasingly narrow distribution; the variance captured by the spatial covariance, and hence $\lambda_1$, decreases accordingly. However, to correctly contextualize the observations above, it is important to keep in mind that although the W matrices are always the same across network variants, their role differs across model variants.

In the Minimal model, $W^{(l)}$ does not enter the recurrent operator: the only term feeding the state back in time is the leak $(1-\gamma^{(l)})h_{t-1}^{(l)}$, while $W^{(l)}$ acts solely on the feedforward drive $x_t^{(l-1)}$. This does not mean that $W^{(l)}$ is irrelevant to the measured timescales — since we estimate them from $r_t^{(l)} = \sigma(h_t^{(l)})$, the learned weights still shape the autocorrelation through the gating of the rectified activity (see below). Specifically, the recurrent dynamics are given by:

$$h_t^{(l)} = W^{(l)} x_t^{(l-1)} + (1-\gamma^{(l)})h_{t-1}^{(l)} + \eta_t^{(l)}, \qquad r_t^{(l)} = \sigma(h_t^l) \tag{1}$$

The istantaneous Jacobian obtained by linearizing the dynamics reads:

$$\frac{\partial h_t^{(l)}}{\partial h_{t-1}^{(l)}} = (1-\gamma^{(l)})I, \qquad J_t^{(r\leftarrow h)} \equiv \frac{\partial r_t^{(l)}}{\partial h_{t-1}^{(l)}} = \frac{\partial r_t^{(l)}}{\partial h_t^{(l)}} \frac{\partial h_t^{(l)}}{\partial h_{t-1}^{(l)}} = D_t^{(l)}(1-\gamma^{(l)}) \tag{2}$$

where $D_t^{(l)} = \mathrm{diag}(\sigma'(h_t^{(l)})) = \mathrm{diag}(\mathbf{1}_{h_t^{(l)}>0})$. This expression makes explicit a separation of roles: the leak factor $(1-\gamma^{(l)})$ sets the maximal linearized persistence of perturbations, while learning affects timescales primarily through the statistics of the gating process, since $D_t^{(l)} = \mathrm{diag}\Big(\mathbf{1}_{W^{(l)}x_t^{(l-1)}+(1-\gamma^{(l)})h_{t-1}^{(l)}+\eta_t^{(l)}>0}\Big)$. Thus, trained vs. random $W^{(l)}$ can strongly change which units remain active over time (and hence which fluctuations contribute to measured intrinsic timescales), even when $\gamma^{(l)}$ is held fixed.

In the Adaptive model, by contrast, $W^{(l)}$ *does* enter the recurrent dynamics directly, through the adaptation feedback $-\beta W^{(l)} s_t^{(l)}$, since the trace $s_t^{(l)}$ integrates past activity $r_{t-1}^{(l)}$. Taking $(h^{(l)}, s^{(l)})$ as the state, the one-step Jacobian reads

$$\mathcal{J}_t^{(l)} = \begin{pmatrix} (1-\gamma^{(l)})\, I - \beta(1-\alpha)\, W^{(l)} D_{t-1}^{(l)} & -\alpha\beta\, W^{(l)} \\ (1-\alpha)\, D_{t-1}^{(l)} & \alpha\, I \end{pmatrix}, \qquad D_{t-1}^{(l)} = \mathrm{diag}(\mathbf{1}_{h_{t-1}^{(l)}>0}). \tag{3}$$

Here the weight matrix appears explicitly in the linearized recurrence, so that analyzing its spectrum becomes pertinent — this is the regime in which the reviewer's dynamical-systems intuition applies, and motivates replicating the randomization analysis for this variant (Suppl. Fig 6.). Two features nonetheless distinguish it from a classical fixed-spectrum recurrent network. First, $W^{(l)}$ enters gated by the trajectory-dependent active set $D_{t-1}^{(l)}$ and scaled by the adaptation strength $\beta$, so its spectral radius does not control the dynamics on its own. Second, the block structure of $\mathcal{J}_t^{(l)}$ carries a slow eigenvalue $\approx \alpha$, associated with the adaptation trace, with timescale $\tau_s \approx 1/(1-\alpha)$ set by $\alpha$ and independent of $W^{(l)}$. The intrinsic-timescale hierarchy thus reflects the joint action of the leak $\gamma^{(l)}$, the adaptation $(\alpha, \beta)$, and the gated weights, rather than the spectrum of $W^{(l)}$ alone.

In summary, the dynamical-systems intuition referred to by the reviewer does not apply to the Minimal model — where the recurrence reduces to the isotropic leak and learning acts only through gating — and is only partially pertinent to the Adaptive model, where $W^{(l)}$ enters the recurrence but gated by $D_{t-1}^{(l)}$ and alongside an adaptation timescale set by $\alpha$. In neither case does a systematic trend in the spectrum of $W^{(l)}$ account by itself for the observed hierarchy of timescales.

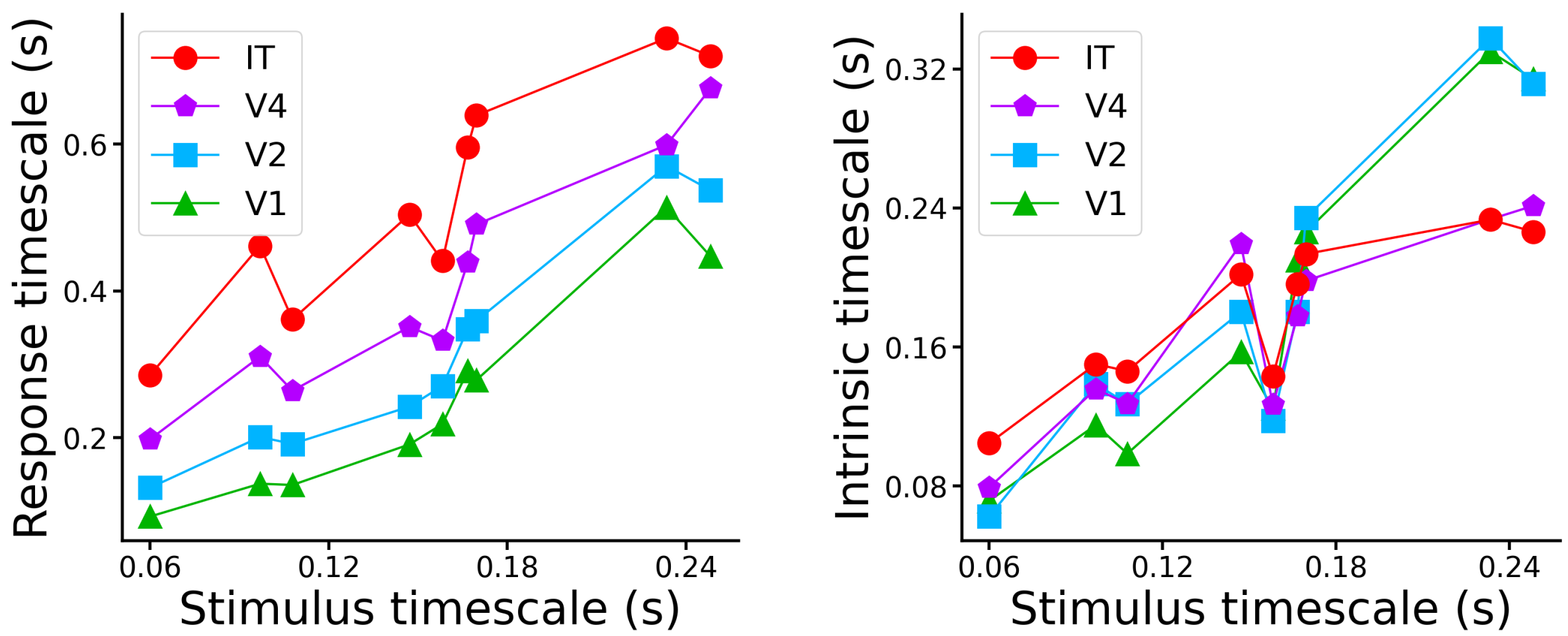


(a) Response timescales adaptive random

(b) Intrinsic timescales adaptive random

### R1.3

> Table 3 shows a drastic drop in performance for the models that successfully reproduce the timescales, in comparison with the original CORnet-RT. The discussion about trade-off between biological realism and functional objective would be helpful.

**Response.** Thanks, we have expanded our discussion of this point, emphasizing that despite a lack of retraining or fine tuning our models still perform better than other, recently published, biologically constrained/inspired recurrent networks for visual object classification (Soo et al. 2024), and that the measured performance drop with respect to CORnet-RT is only a conservative upper bound, as unlike CORnet-RT our models are not trained end-to-end to minimize classification error.

> The Methods state noise is added "in the second convolutional layer of each area". The equations describe noise $\eta_t$ added to the hidden state. It would be useful to clarify if it is added to the input of the recurrence or the output.

**Response.** We thank the reviewer for spotting this ambiguity. The noise is added to the hidden (pre-activation) state $h_t^{(l)}$ after the second convolution and normalization, together with the leak term, and therefore enters the recurrent loop; it is not injected inside the convolution nor added to the post-activation output $r_t^{(l)}$. This is consistent with all the update equations, where $\eta_t^{(l)}$ is summed onto $h_t^{(l)}$ rather than appearing inside the argument of $W$. We have rephrased the corresponding Methods passage to remove the ambiguity, and we now also refer the reader to the area circuit diagram Suppl. Fig.5.

## Reviewer #2

> In their manuscript, the authors disentangle how systematic manipulations to CORnet can result in response and intrinsic timescales across the network hierarchy compatible with experimental results from the visual cortex of rodents. The paper is very well written with clear illustrations and interesting conclusions. I have a few comments, however, that the authors may want to consider.

**Response.** We thank the Reviewer for their positive assessment. We address individual points below.

### R2.1

> response vs. intrinsic timescales: The authors did a great effort in defining their timescales. In the context of comp.neuro this is indeed a bit unsorted. However, this also introduces some constraints that may require a bit more of discussion. Specifically, I would associate a response timescale with the time-to-response of a stimulus. Here, however, the response timescale is related to the autocorrelation time of the trial-mean corrected population response. This is connected to correlations from recurrent activity, cf. the fading effect of perturbations defined by H. Jäger (https://www.ai.rug.nl/minds/uploads/EchoStatesTechRep.pdf) or the concept of recurrency (https://www.pnas.org/doi/pdf/10.1073/pnas.1818972116) when combined with time-dependent ensemble mean (https://journals.aps.org/pre/abstract/10.1103/PhysRevE.102.040301). I appreciate the clear definitions provided and understand that they follow previous work, but I believe that it could be made more explicitly how the results add to the broader concepts of timescales from recurrent dynamics. Specifically, the way I understand it is that the intrinsic and response timescales should be the same for spontaneous activity where the ensemble mean is stationary and what should have been the reason why Murray et al. used the pre-stim trials with single-unit autocorrelation analyses.

**Response.** Thank you – indeed, there are other ways of defining the characteristic time scales in neural activity, which we did not engage with in our initial submission. We do so now, by better tying into the literature cited by the Reviewer. Regarding the relationship between response and intrinsic timescales, yes, in absence of stimulus assuming that the ensemble mean is stationary, the two measures coincide. However, when the response is not stationary the correlations from which the two types of timescales are computed are analogous to 'signal' and 'noise' correlations (with distinct timepoints playing the role of distinct stimuli in the classical definition of signal and noise correlations). This point overlaps also with point 2 by R3. We now underscore better the connection between our timescale definitions and the classical concepts of signal and noise correlations in the text.

### R2.2

> Correlation analysis: This here considers the full vector of trials, does this mean the covariance across trials is included in the analysis or is it only the temporal covariance that remains? From Eq. 15 I would have expected X to be a random scalar, which I associated to a pixel in 1) and to a neuron in 2) and which I would have thought were averaged later. But maybe I did get this completely wrong.

**Response.** We apologize for the confusion. In all three cases described in the section "Estimation of temporal correlation", $X_t$ is indeed, conceptually, a random scalar (so that $\mathrm{Var}[X_t]$ and $\mathrm{Cov}[X(t), X(t+\tau)]$ are also scalars). The reason why in the text we talk about vectors is simply that we think of the set of samples for these random scalars as being organized as vectors. For the movie stimuli (point 1) the random scalar $X_t$ is the intensity of a pixel across the image frame at a fixed time t, and the vector is the (unrolled) image frame. For the stimulus-driven neural activity, the scalar is the value of the (time-centered) population PSTH across the population, at a fixed time t, and the vector is the *t-th* time slice of the population PSTH. For the intrinsic neuronal activity, the random scalar is the activity of a fixed neuron at a fixed time t across all trials, and the vector contains all available samples of this activity (so one entry per trial). We have updated the text to clarify this passage.

**R2.3**

Timescales from recurrent dynamics: The authors rightfully identified that a leak timescale can prolong timescales. In my experience, however, the largest effort in the field of theoretical neuroscience is devoted to how population timescales can emerge beyond the single-unit leak timescale. Examples include (https://journals.aps.org/prx/abstract/10.1103/PhysRevX.5.041030) or (https://www.frontiersin.org/journals/systems-neuroscience/articles/10.3389/fnsys.2018.00055/full). The core concept is again that of recurrent dynamics (see above examples). In the context of the present results, this would imply that what changes is the eigenvalue of the weight matrix (W) with fixed gamma across layers. Consequently, it would be interesting to calculate the eigenvalues of the trained weights, check if there is a trend, and keep that trend in the minimal model. However, one could also argue that the gamma is already the effective timescale of a local population of neurons (here modeled by a rate code). What I want to aim at here is that in either case, it would be helpful that the results are related to the current concepts in theoretical neuroscience, possibly within the discussion.

**Response.** Thank you. This is a very insightful point which overlaps with point 2 by R1. Please see our response above where we discuss the spectral properties of the connectivity matrices. Moreover, we agree that the leak parameter $\gamma$ can be interpreted as controlling an effective population level timescale. In fact, similar mechanisms appear in clustered or self-coupled random network models, where individual units represent local neuronal assemblies and a self-coupling parameter controls the intrinsic persistence of activity within each cluster (Stern, Istrate, and Mazzucato 2023). In such frameworks, increasing the self-coupling strength directly prolongs the local timescale without necessarily requiring the network to operate at a global critical point. At the same time, the theoretical works cited by the reviewer primarily analyze randomly connected recurrent networks to uncover universal dynamical regimes (e.g., transitions to chaos or reverberating states). These models aim to characterize generic properties of recurrent circuits under random connectivity assumptions. In contrast, our work focuses on hierarchical networks whose connectivity arises from supervised training for visual recognition. While such models have traditionally emphasized feedforward representations and static selectivity, the role of intrinsic temporal dynamics has received comparatively less attention. Our goal is to bridge these perspectives by examining how temporal persistence and intrinsic timescales manifest in structured, task-optimized architectures. We have now updated the text and the figures to include the spectral analysis and to help the reader better connect our results with established results from the theory of recurrent networks. We also expanded this point in our discussion to better frame how this work contributes with respect to cited references.

**R2.4**

The claim "the network determinants of these timescale hierarchies are not well understood in realistic systems ... " is not well justified in the light of the above examples, specifically we recent new analyses such as (https://journals.plos.org/ploscompbiol/article?id=10.1371/journal.pcbi.1012355) or (https://www.pnas.org/doi/full/10.1073/pnas.2208998120) that look at timescales or eigenvalues along the hierarchy in models with noise.

**Response.** By "realistic" here we meant specifically image-computable, which is a property that distinguishes our models from the ones cited by the reviewer. We realize that this was a misleading choice of words, and have now clarified this in the text. We have also made sure to refer to both papers mentioned by the reviewer in the text.

### R2.5

Mechanism of the presented results: Overall, I find the results very interesting and I believe consistent with my expectations from theoretical neuroscience. What I am lacking is more insight into how the results come about. The organization proceeds by introducing a model, showing a Figure (very nice consistent layout! I would suggest, however, to fix all x and y scales to the same range) and then relating that to Fig. 1. However, a mechanism why the model agrees more or less is not provided. I understand that this cannot be done extensively, but simple checks like Eigenvalues of the recurrent interactions or relation to the timescales of the leak would have been nice.

**Response.** Here also we refer the Reviewer to our response to R1. Moreover, besides discussing more extensively the spectra of the connectivity matrices we now also show the effect of different choices of leak rate on the timescales (Suppl. Fig 2-3), highlighting that even setting the leak to be the same across all layers still results in a more ordered progression of timescales than what we observe when randomizing the feedforward prediction weights (while still keeping $\gamma$ to our original values), Fig 7. This underscores how our findings are due to the interaction between (learned) feedforward processing, integration (leak), and recurrent/adaptive processing and can't be trivially explained by any one factor. Teasing out the exact nature of this interaction is a promising area of research for future work.

Thank you also for the suggestion about the scale of the plots. We have made it uniform across the stochastic, adaptive, and minimal network variants, as these could all easily be visualized using the same axis limits. We have left custom limits for the other variants, as we found that imposing the same limits on all plots would hinder readability.

### R2.6

Model parameter: I wonder if it helps to directly introduce timescales instead of the parameters gamma, alpha. Specifically Eq. (7) can be related to an exponential integration with a given timescale. This would possibly allow to explain increases in the response timescales "trivially".

**Response.** This is an interesting suggestion, but we believe that the role of gamma and alpha in explaining "trivially" our results is addressed more directly by the new analyses we have performed that show the effect of changing these parameters and the associated text. Moreover, even notationally, introducing two more timescales in the paper (the ones associated trivially with gamma and alpha) would in our opinion risk confusing the reader.

### R2.7

Models: I think I am lacking one detail about the model. Specifically, I fail to see what $x_t$ is for higher areas. Naively, I would have expected it to be $r_t$ (the nonlinear version of $h_t$) but this is the recurrent part. From the figure, it seems like h is passed up, but why is there no nonlinearity applied then. Also, why would it be $x_t$ and not $x_{t-1}$. I guess this is a misunderstanding on my side that originates from presenting the model very well in a simplified manner and may be clarified easily.

**Response.** We apologize for the confusion. Indeed it is r that is passed "up" to the next area; h is passed "on" to the next time step in the same area. We have added a new schematic in Supplementary Figure 5 that illustrate more in detail how activity is passed up from one layer to the next across two time steps.

### R2.8

> Noise: In the model section, we define $\sigma = 0.7 * \alpha$ which introduces two issues: i) why would we do that and not just change $\sigma$ and ii) there is an overlap with the $\boldsymbol{\alpha}$ from Table 6. Maybe sticking to $\sigma$ would be sufficient unless the 0.7 changes across layers (which I would not expect from the main text).

**Response.** Good catch. We have updated the text to use only $\sigma$.

### R2.9

> Stimuli: The phase-scrambled movies I assume were obtained from shuffling the frequencies in Fourier space, but the sentence in line 370 does not tell us.

**Response.** Yes, they were obtained by shuffling the phases of the Fourier transform of the video. We now mention this explicitly in the text and point the reader to the Methods section of the paper where the stimulus was defined for more detail.

## Reviewer #3

> In this study, the authors investigate the mechanism required to explain the hierarchy of neural timescales observed in the rat brain. For this purpose, they use a previously developed CNN models of the ventral stream that integrates convolution and recurrence in its architecture and show that the implemented spatial invariance in this model is not sufficient to capture the hierarchy of timescales. Then, they show that including leaky integration and adaptation can introduce a hierarchical structure in network dynamics.
> The study is interesting, and the details are presented clearly. However, I have several major comments regarding the main claims of the study listed below.

### R3.1

> Biological plausibility of CORnet-RT and generalizability of the results: CORnet-RT indeed has a higher brainscore than conventional CNNs, but the brainscore evaluates the time-averaged neural responses and not actual dynamics. The fact that the baseline CORnet-RT does not show a hierarchy of timescales and it is required to add more biological mechanisms, such as leaky integration and adaptation, is not surprising. Therefore, the claim that "intrinsic timescales depend on the details of the functions implemented on each layer" is very specific to this model.

**Response.** Thanks for pointing this out. Indeed, that part of sentence as quoted by the Reviewer reads as potentially misleading. What we are trying to say in that passage is not that it is remarkable that the specific values of the intrinsic timescales depend on the functions implemented by the network – this is hardly surprising – but that their hierarchical organization does, in contrast with the hierarchical organization of the response timescales (which does not depend on such details). This is easier to understand if one considers the full sentence, which says "We show that, surprisingly, the organization of the representation timescales is set by the broad architectural features of the network, regardless of training, while the intrinsic timescales depend on the details of the functions implemented on each layer". However, we agree that our original wording was confusing, and we have updated it to make it clearer.

Additionally, stating that "the network determinants of these timescale hierarchies are not understood in realistic systems, as the classical theory is based on models without noise, recurrence, or adaptive mechanisms" is not true, considering the extensive literature of previous models built to explain the hierarchy of timescales. In fact, more biologically realistic models of primate and rodent brain that include noise and recurrence show that incorporating simple biological gradients is sufficient to capture the hierarchy of intrinsic timescales (Chaudhuri et al., 2015, Neuron) and their modulation during task (Wang et al., 2024, arXiv; Ding et al., 2024, eLife), without explicit task training. Therefore, it is important to avoid general claims that might be limited to this model, especially in the abstract. The authors also need to discuss their findings in the context of these prior studies, as well as the biological relevance of the mechanisms they propose.

**Response.** Good point – this is something that R2 also picked up on. Here we meant to say that timescale hierarchies are not well understood (or even studied) in image-computable models of the class that has been used, since the early works by Yamins, to predict the actual response properties of individual neurons in the ventral stream. We have amended our wording in the text to express this better and expanded our discussion section to provide a better comparison of our results in the context of prior studies.

### R3.2

Intrinsic timescales: - Definition: I was quite surprised that intrinsic timescales measured from the rat data (Fig 1b) depend on the stimulus timescale, especially given the definition provided in the manuscript: "intrinsic timescales reflect the duration over which neural activity remains temporally correlated in the absence of stimulus variability". If the timescales do depend on stimulus features, the term "intrinsic" is not appropriate, especially when it is measured from stimulus-driven activity.

**Response.** We understand the Reviewer's surprise in coming across the phenomenon that intrinsic timescales, as measured in real data, can weakly depend on the properties of the input. However, we disagree that the term "intrinsic" is not appropriate. Intrinsic timescales, as we define them, measure the timescale of noise correlations, in the classic meaning established for over thirty years (Gawne and Richmond 1993; Panzeri et al. 1999; Averbeck, Latham, and Pouget 2006; Kohn, Coen-Cagli, et al. 2016), when these are computed across different time points instead of different neurons. Noise correlations were introduced to describe trial-by-trial fluctuations of neural responses evaluated at fixed stimulus, therefore capturing a property of the neural response that is to a first approximation unrelated to the signal that is meant to be conveyed. Hence their name, and their introduction in contrast with "signal" correlations (Gawne and Richmond 1993). However, it is well known that noise correlations can in fact depend on the stimuli (see for instance Kohn and Smith 2005; Ponce-Alvarez et al. 2013; Franke et al. 2016; Zylberberg et al. 2016 or on the brain state (e.g. Cohen and Maunsell 2009), and that their impact on information content of a neural code can depend strongly on the way in which they depend on the stimulus (e.g. Josić et al. 2009; Moreno-Bote et al. 2014). Importantly, temporal noise correlations were also found to vary depending on the brain state, behavioral factors, or properties of the input (Runyan et al. 2017; Valente et al. 2021), a finding that aligns fully with the report in Piasini et al. 2021 that intrinsic timescales can also depend on the stimulus. So in summary, (1) we are not introducing this usage of the expression "intrinsic timescales" in this work, but rather using it consistently with previous work (Piasini et al. 2021), and changing it would introduce confusion; (2) in any case, we believe this established usage of the expression to be perfectly reasonable by analogy to the notion of noise correlations, where research has shown that second-order statistics of neural responses

can depend on the stimulus and other factors. However, we thank the reviewer for pointing out that the data in Figure 1b may be somewhat counterintuitive for part of the readership. We have modified the text to underscore the connection of our timescales to signal and noise correlations and to give further pointers to the reader.

> Estimation: For estimating intrinsic timescales from the CORnet-RT model, it would be more reasonable to use an uncorrelated noisy input (mimicking spontaneous neural activity) instead of movies. This is particularly important given the fact that in some figures (e.g., Leaky CORnet in Fig 4), a hierarchy of timescales is observed for some stimulus timescales and not others, and the authors have currently discarded these results.

**Response.** This partly overlaps with the previous point, so see our answer above for the reasoning behind measuring and reporting intrinsic timescales across a variety of stimuli. Moreover, in practice we already perform the requested control, because our "white noise" stimulus is a totally uncorrelated stimulus. The timescale hierarchy results, restricted to only the responses to this stimulus across network variants, broadly recapitulate what we report for other stimuli. Finally, however, it is not clear to us in what sense uncorrelated noisy input would mimic spontaneous neural activity. The input to the network represents visual stimuli, so it is not obvious that spontaneous activity should correspond to the response to uncorrelated stimuli. Therefore, in summary, we are happy to report that restricting our analysis to only uncorrelated visual stimulus reproduces our main findings, although we don't think that a specific connection should be drawn between this result and spontaneous activity.

### R3.3

> Estimation of timescales from the model: - The details of timescale estimation from CORnet-RT activity need to be clarified further in the methods. Given that CORnet-RT dynamics evolve on discrete time steps, how were they compared to spiking activity in seconds? How is the time discretization step defined, and how does it affect the results?

**Response.** Thank you for highlighting this important point. The bridge between the networks' discrete time and the time of neural activity, measured in seconds, comes from the framerate of the video stimuli (30fps, with the exception of the white noise stimulus that for the neural recordings was played at 20fps), which are common between the rat experiments and our computational analyses, together with the assumption that our networks process one video frame per discrete time step. This, in turn, results in a step duration of 33ms, which matches the bin size used for the original analyses of the spiking data, and is a common bin/time step size for analyses of cortical spiking activity. These aspects were mentioned only briefly in the Methods in our initial submission (and the connection with the bin size of the neural data analysis was not mentioned at all); we have emphasized them better in our revised text.

> It seems that CORnet-RT dynamics show an oscillatory component (based on the fitted function to autocorrelations). It would be interesting to report how the frequency of these oscillations changes across layers/models and also show some example autocorrelations of their behavior. Also, do authors observe a similar oscillation in neural activity?

**Response.** Yes, our choice of functional form for fitting the correlation functions mirrors what was done in the electrophysiology study, as the oscillatory components of the autocorrelations were observed in both cases. For the autocorrelation functions of the electrophysiology data, we refer the Reviewer to figure 4 in Piasini et al. 2021. We have added example autocorrelations from our simulations and a table with the values of the oscillation frequencies in Figure 9.

### R3.4

> Dependence of results on hyperparameters: Models with leak and adaptation require a set of hyperparameters that seem to be manually chosen. It is important to show how the presented results depend on the choice of hyperparameters $(\gamma, \alpha, \beta)$

**Response.** We have performed a robustness analysis, showing that qualitatively our results remain valid over a broad range of the values of the hyperparameters. We report this analysis in supplementary figures 2-3-4, and comment on them in additional text in the supplementary material. The robustness analysis is also informative in showing that the most prominent failure mode of the model happens when $\beta$ (governing the intensity of adaptation) grows beyond a certain threshold. In this scenario, the biological plausibility of the model begins to break down, as strong low-frequency modulations of the firing rates begin to emerge leading to correlations that persist at very high values for long lags. This is markedly different from the behavior observed in the neural recordings (compare supplementary figure 4 and Figure 4 in Piasini et al. 2021). Therefore, the results on the timescale hierarchies are robust in the sense that they are valid for ranges of the hyperparameters such that, when they are exceeded, the network begins to behave in markedly unplausible ways rather than simply exhibiting different timescale rankings.

### R3.5

> Given the computational nature of this study, it is important that the code for the model simulations and analyses be made available for the review process (and after publication to other readers for reproducibility).

**Response.** We agree. The code is now publicly available on github at github.com/Saravaretti/hierarchical-cnn-rnn.

#### Minor comments

> What is the logic behind resampling a subset of neurons for computing timescales? To show the variability, it would have been more reasonable to train CORnet-RT with multiple seeds and perform analyses across multiple trained networks (but this might be beyond the scope of this study).

**Response.** We agree that in principle training CORnet-RT with multiple seed would be a good way of assessing variability. However, as the Reviewer guessed, it would be out of the scope of the present paper due to computational cost. Therefore, we adopted the "neural population subsampling" approach, which is commonly used in studies of deep neural networks for computational vision neuroscience (Cadieu et al. 2014; Bashivan, Kar, and DiCarlo 2019; Muratore et al. 2022; Galella, Wehrheim, and Kaschube 2025).

> The role and location of nonlinearity in different models need to be clarified in the manuscript. For example, for the Minimal Model, where $r_t$ is not used in $h_t$ equation (eq. 10, 11), how does nonlinearity affect the dynamics? Does it affect transition of activity between layers?

**Response.** In short, yes, the signal that is "passed up" from one layer to the next always goes through the nonlinearity, for all network variants. More broadly, this point by the Reviewer partly overlaps with point 7 by R2, underscoring that our description of the network dynamics was not sufficiently clear. We have now clarified this in the text, and added a schematic that

shows in detail how the signal is processed between two layers and two timesteps (Supplementary Figure 4).

## References


Averbeck, Bruno B., Peter E. Latham, and Alexandre Pouget (May 2006). “Neural Correlations, Population Coding and Computation”. In: *Nature Reviews Neuroscience* 7.5, pp. 358–366. ISSN: 1471-0048. DOI: 10.1038/nrn1888. URL: https://www.nature.com/articles/nrn1888 (visited on 07/26/2024).

Bashivan, Pouya, Kohitij Kar, and James J. DiCarlo (May 2019). “Neural Population Control via Deep Image Synthesis”. In: *Science* 364.6439, eaav9436. ISSN: 0036-8075, 1095-9203. DOI: 10.1126/science.aav9436. (Visited on 01/17/2022).

Cadieu, Charles F. et al. (2014). “Deep neural networks rival the representation of primate IT cortex for core visual object recognition”. In: *PLoS Comput Biol* 10.12, e1003963. DOI: 10.1371/journal.pcbi.1003963.

Cohen, Marlene R. and John H. R. Maunsell (Dec. 2009). “Attention Improves Performance Primarily by Reducing Interneuronal Correlations”. In: *Nature Neuroscience* 12.12 (12), pp. 1594–1600. ISSN: 1546-1726. DOI: 10.1038/nn.2439. URL: https://www.nature.com/articles/nn.2439 (visited on 03/23/2022).

Franke, Felix et al. (Jan. 20, 2016). “Structures of Neural Correlation and How They Favor Coding”. In: *Neuron* 89.2, pp. 409–422. ISSN: 0896-6273. DOI: 10.1016/j.neuron.2015.12.037. URL: https://www.cell.com/neuron/abstract/S0896-6273(15)01139-3 (visited on 06/25/2026).

Galella, Santiago, Maren Wehrheim, and Matthias Kaschube (Dec. 4, 2025). “Dimensionality Mismatch Between Brains and Artificial Neural Networks”. In: *Advances in Neural Information Processing Systems*.

Gawne, T. J. and B. J. Richmond (July 1, 1993). “How Independent Are the Messages Carried by Adjacent Inferior Temporal Cortical Neurons?” In: *Journal of Neuroscience* 13.7, pp. 2758–2771. ISSN: 0270-6474, 1529-2401. DOI: 10.1523/JNEUROSCI.13-07-02758.1993. PMID: 8331371. URL: https://www.jneurosci.org/content/13/7/2758 (visited on 05/29/2026).

He, Kaiming et al. (Dec. 2015). “Delving Deep into Rectifiers: Surpassing Human-Level Performance on ImageNet Classification”. In: *2015 IEEE International Conference on Computer Vision (ICCV)*. 2015 IEEE International Conference on Computer Vision (ICCV). Santiago, Chile: IEEE, pp. 1026–1034. ISBN: 978-1-4673-8391-2. DOI: 10.1109/ICCV.2015.123. URL: http://ieeexplore.ieee.org/document/7410480/ (visited on 06/25/2026).

Josić, Krešimir et al. (Oct. 1, 2009). “Stimulus-Dependent Correlations and Population Codes”. In: *Neural Computation* 21.10, pp. 2774–2804. ISSN: 0899-7667. DOI: 10.1162/neco.2009.10-08-879. URL: https://doi.org/10.1162/neco.2009.10-08-879 (visited on 06/25/2026).

Kohn, Adam, Ruben Coen-Cagli, et al. (2016). “Correlations and Neuronal Population Information”. In: *Annual Review of Neuroscience* 39.1, pp. 237–256. DOI: 10.1146/annurev-neuro-070815-013851. PMID: 27145916. URL: https://doi.org/10.1146/annurev-neuro-070815-013851 (visited on 04/05/2022).

Kohn, Adam and Matthew A. Smith (Apr. 6, 2005). “Stimulus Dependence of Neuronal Correlation in Primary Visual Cortex of the Macaque”. In: *Journal of Neuroscience* 25.14, pp. 3661–3673. ISSN: 0270-6474, 1529-2401. DOI: 10.1523/JNEUROSCI.5106-04.2005. PMID: 15814797. URL: https://www.jneurosci.org/content/25/14/3661 (visited on 06/25/2026).

Moreno-Bote, Rubén et al. (Oct. 2014). “Information-Limiting Correlations”. In: *Nature Neuroscience* 17.10 (10), pp. 1410–1417. ISSN: 1546-1726. DOI: 10.1038/nn.3807. URL: https://www.nature.com/articles/nn.3807 (visited on 03/23/2022).

Muratore, Paolo et al. (Dec. 6, 2022). "Prune and Distill: Similar Reformatting of Image Information along Rat Visual Cortex and Deep Neural Networks". In: *Advances in Neural Information Processing Systems* 35, pp. 30206–30218. URL: https://proceedings.neurips.cc/paper_files/paper/2022/hash/c2d82a425af4c18a35049899fea5ee82-Abstract-Conference.html (visited on 05/08/2023).

Panzeri, Stefano et al. (May 22, 1999). "Correlations and the Encoding of Information in the Nervous System". In: *Proceedings of the Royal Society B: Biological Sciences* 266.1423, pp. 1001–1012. ISSN: 0962-8452. DOI: 10.1098/rspb.1999.0736. URL: https://doi.org/10.1098/rspb.1999.0736 (visited on 05/29/2026).

Piasini, Eugenio et al. (2021). "Temporal stability of stimulus representation increases along rodent visual cortical hierarchies". In: *Nat Commun* 12, p. 4448. DOI: 10.1038/s41467-021-24456-3.

Ponce-Alvarez, Adrián et al. (Aug. 6, 2013). "Stimulus-Dependent Variability and Noise Correlations in Cortical MT Neurons". In: *Proceedings of the National Academy of Sciences* 110.32, pp. 13162–13167. DOI: 10.1073/pnas.1300098110. URL: https://www.pnas.org/doi/abs/10.1073/pnas.1300098110 (visited on 06/25/2026).

Runyan, Caroline A. et al. (2017). "Distinct timescales of population coding across cortex". In: *Nature* 548.7665, pp. 92–96. DOI: 10.1038/nature23020.

Soo, Wayne W. M. et al. (2024). "Recurrent Neural Network Dynamical Systems for Biological Vision". In: *Advances in Neural Information Processing Systems*. Vol. 37. Curran Associates, Inc. URL: https://proceedings.neurips.cc/paper_files/paper/2024/hash/f536d5697b79a9b3b3debbb7a552a7da-Abstract-Conference.html.

Stern, Merav, Nicolae Istrate, and Luca Mazzucato (2023). "A reservoir of timescales emerges in recurrent circuits with heterogeneous neural assemblies". In: *eLife* 12, e86552. DOI: 10.7554/eLife.86552.

Valente, Martina et al. (July 2021). "Correlations Enhance the Behavioral Readout of Neural Population Activity in Association Cortex". In: *Nature Neuroscience* 24.7, pp. 975–986. ISSN: 1546-1726. DOI: 10.1038/s41593-021-00845-1. (Visited on 03/28/2022).

Zylberberg, Joel et al. (Jan. 20, 2016). "Direction-Selective Circuits Shape Noise to Ensure a Precise Population Code". In: *Neuron* 89.2, pp. 369–383. ISSN: 0896-6273. DOI: 10.1016/j.neuron.2015.11.019. URL: https://www.cell.com/neuron/abstract/S0896-6273(15)01025-9 (visited on 06/25/2026).
