# Supplementary material for: Noisy models of the ventral stream reveal the impact of recurrence and learned representations on information processing timescales
Source: PLoS Comput Biol. 2026 Aug 27;22(8):e1014653. doi: 10.1371/journal.pcbi.1014653 (PMC13541256; doi:10.1371/journal.pcbi.1014653)
Supplement: Peer review history

# Peer review: Decision Letter 0

**DOI:** 10.1371/journal.pcbi.1014653.r001

**Authors:** Tianming Yang, Yuanning Li

3 Feb 2026

Noisy models of the ventral stream reveal the impact of recurrence and learned representations on information processing timescales

PLOS Computational Biology

Dear Dr. Piasini,

Thank you for submitting your manuscript to PLOS Computational Biology. After careful consideration, we feel that it has merit but does not fully meet PLOS Computational Biology's publication criteria as it currently stands. Therefore, we invite you to submit a revised version of the manuscript that addresses the points raised during the review process.

Please submit your revised manuscript by Apr 05 2026 11:59PM. If you will need more time than this to complete your revisions, please reply to this message or contact the journal office at ploscompbiol@plos.org. When you're ready to submit your revision, log on to https://www.editorialmanager.com/pcompbiol/ and select the 'Submissions Needing Revision' folder to locate your manuscript file.

Please include the following items when submitting your revised manuscript:

* A letter that responds to each point raised by the editor and reviewer(s). You should upload this letter as a separate file labeled 'Response to Reviewers'. This file does not need to include responses to formatting updates and technical items listed in the 'Journal Requirements' section below.

* A marked-up copy of your manuscript that highlights changes made to the original version. You should upload this as a separate file labeled 'Revised Manuscript with Track Changes'.

* An unmarked version of your revised paper without tracked changes. You should upload this as a separate file labeled 'Manuscript'.

If you would like to make changes to your financial disclosure, competing interests statement, or data availability statement, please make these updates within the submission form at the time of resubmission. Guidelines for resubmitting your figure files are available below the reviewer comments at the end of this letter

We look forward to receiving your revised manuscript.

Kind regards,

Yuanning Li

Academic Editor

PLOS Computational Biology

Tianming Yang

Section Editor

PLOS Computational Biology

**Journal Requirements:**

If the reviewer comments include a recommendation to cite specific previously published works, please review and evaluate these publications to determine whether they are relevant and should be cited. There is no requirement to cite these works unless the editor has indicated otherwise.

1) We ask that a manuscript source file is provided at Revision. Please upload your manuscript file as a .doc, .docx, .rtf or .tex. If you are providing a .tex file, please upload it under the item type u2018LaTeX Source Fileu2019 and leave your .pdf version as the item type u2018Manuscriptu2019.

2) Please upload all main figures as separate Figure files in .tif or .eps format. For more information about how to convert and format your figure files please see our guidelines:

https://journals.plos.org/ploscompbiol/s/figures

3) We notice that your supplementary Figures are included in the manuscript file. Please remove them and upload them with the file type 'Supporting Information'. Please ensure that each Supporting Information file has a legend listed in the manuscript after the references list.

4) Some material included in your submission may be copyrighted. According to PLOSu2019s copyright policy, authors who use figures or other material (e.g., graphics, clipart, maps) from another author or copyright holder must demonstrate or obtain permission to publish this material under the Creative Commons Attribution 4.0 International (CC BY 4.0) License used by PLOS journals. Please closely review the details of PLOSu2019s copyright requirements here: PLOS Licenses and Copyright. If you need to request permissions from a copyright holder, you may use PLOS's Copyright Content Permission form.

Please respond directly to this email and provide any known details concerning your material's license terms and permissions required for reuse, even if you have not yet obtained copyright permissions or are unsure of your material's copyright compatibility. Once you have responded and addressed all other outstanding technical requirements, you may resubmit your manuscript within Editorial Manager.

Potential Copyright Issues:

i) Please confirm (a) that you are the photographer of 2, or (b) provide written permission from the photographer to publish the photo(s) under our CC BY 4.0 license.

5) In the online submission form, you indicated that The code used to generate the results and figures in this paper will be made available upon request.. All PLOS journals now require all data underlying the findings described in their manuscript to be freely available to other researchers, either

1. In a public repository

2. Within the manuscript itself

3. Uploaded as supplementary information.

This policy applies to all data except where public deposition would breach compliance with the protocol approved by your research ethics board. If your data cannot be made publicly available for ethical or legal reasons (e.g., public availability would compromise patient privacy), please explain your reasons by return email and your exemption request will be escalated to the editor for approval. Your exemption request will be handled independently and will not hold up the peer review process, but will need to be resolved should your manuscript be accepted for publication. One of the Editorial team will then be in touch if there are any issues.

6) Please amend your detailed Financial Disclosure statement. This is published with the article. It must therefore be completed in full sentences and contain the exact wording you wish to be published.

1) State the initials, alongside each funding source, of each author to receive each grant. For example: "This work was supported by the National Institutes of Health (####### to AM; ###### to CJ) and the National Science Foundation (###### to AM)."

2) State what role the funders took in the study. If the funders had no role in your study, please state: "The funders had no role in study design, data collection and analysis, decision to publish, or preparation of the manuscript."

3) If any authors received a salary from any of your funders, please state which authors and which funders..

If you did not receive any funding for this study, please simply state: u201cThe authors received no specific funding for this work.u201d

7)  Please ensure that the funders and grant numbers match between the Financial Disclosure field and the Funding Information tab in your submission form. Note that the funders must be provided in the same order in both places as well.

**Reviewers' comments:**

Reviewer's Responses to Questions

**Comments to the Authors:**

**Please note here if the review is uploaded as an attachment.**

Reviewer #1: This manuscript studies the network mechanisms behind the hierarchical organization of temporal timescales in the visual ventral stream. Inspired by recent experimental results in rodent visual cortex, the authors try to reproduce the finding that both “response timescales” (the decay of trial-averaged activity) and “intrinsic timescales” (the decay of noise correlations) gradually increase along the anatomical hierarchy. The authors use several modified versions of CORnet-RT (Stochastic, Leaky, Adaptive, Minimal, and Random) to separately examine the roles of recurrence, spatial pooling, intrinsic cellular memory (leaky integration), and learning. Overall, these model variants are well-designed and well-suited for exploring different possible mechanisms.

One important result is the clear separation between the two types of timescales. The authors show that response timescales and intrinsic timescales depend on different factors. Response timescales naturally arise from the deep convolutional structure, while intrinsic timescales do not. In addition, results from the stochastic CORnet suggest that spatial invariance and standard recurrence alone are not enough to produce a hierarchical organization of intrinsic timescales. Instead, explicit leaky integration or adaptation, together with heterogeneous time constants across layers, is required. Another key result concerns learning. Leaky integration by itself is not sufficient; it needs to be combined with learned representations. When the network weights are randomized, the hierarchy of intrinsic timescales disappears, even though the hierarchical leak constants are still present.

Overall, this is a timely and interesting study that helps bridge the gap between static deep learning models of vision and the dynamic, temporally structured activity seen in biological neural circuits. The paper is suitable for acceptance, but there are still some concerns:

1. The “Minimal Model” depends on manually setting the leaky parameter γ to decrease monotonically. While the result that this alone is insufficient without learning is insightful, the claim that the hierarchy “emerges” in the Minimal Model may be somewhat overstated.

2. The novel finding “learned feature selectivity interacts nontrivially with internal dynamics” would be strengthened, by including a spectral analysis of the recurrent weight matrices or the Jacobian of the dynamics for the Trained and Random models”.

3. Table 3 shows a drastic drop in performance for the models that successfully reproduce the timescales, in comparison with the original CORnet-RT. The discussion about trade-off between biological realism and functional objective would be helpful.

4. The Methods state noise is added “in the second convolutional layer of each area”. The equations describe noise η_t added to the hidden state. It would be useful to clarify if it is added to the input of the recurrence or the output.

Reviewer #2: In their manuscript, the authors disentangle how systematic manipulations to CORnet can result in response and intrinsic timescales across the network hierarchy compatible with experimental results from the visual cortex of rodents. The paper is very well written with clear illustrations and interesting conclusions. I have a few comments, however, that the authors may want to consider.

# Comments

- response vs. intrinsic timescales: The authors did a great effort in defining their timescales. In the context of comp.neuro this is indeed a bit unsorted. However, this also introduces some constraints that may require a bit more of discussion. Specifically, I would associate a response timescale with the time-to-response of a stimulus. Here, however, the response timescale is related to the autocorrelation time of the trial-mean corrected population response. This is connected to correlations from recurrent activity, cf. the fading effect of perturbations defined by H. Jäger (https://www.ai.rug.nl/minds/uploads/EchoStatesTechRep.pdf) or the concept of recurrency (https://www.pnas.org/doi/pdf/10.1073/pnas.1818972116) when combined with time-dependent ensemble mean (https://journals.aps.org/pre/abstract/10.1103/PhysRevE.102.040301). I appreciate the clear definitions provided and understand that they follow previous work, but I believe that it could be made more explicitly how the results add to the broader concepts of timescales from recurrent dynamics. Specifically, the way I understand it is that the intrinsic and response timescales should be the same for spontaneous activity where the ensemble mean is stationary and what should have been the reason why Murray et al. used the pre-stim trials with single-unit autocorrelation analyses.

- Correlation analysis: This here considers the full vector of trials, does this mean the covariance across trials is included in the analysis or is it only the temporal covariance that remains? From Eq. 15 I would have expected X to be a random scalar, which I associated to a pixel in 1) and to a neuron in 2) and which I would have thought were averaged later. But maybe I did get this completely wrong.

- Timescales from recurrent dynamics: The authors rightfully identified that a leak timescale can prolong timescales. In my experience, however, the largest effort in the field of theoretical neuroscience is devoted to how population timescales can emerge beyond the single-unit leak timescale. Examples include (https://journals.aps.org/prx/abstract/10.1103/PhysRevX.5.041030) or (https://www.frontiersin.org/journals/systems-neuroscience/articles/10.3389/fnsys.2018.00055/full). The core concept is again that of recurrent dynamics (see above examples). In the context of the present results, this would imply that what changes is the eigenvalue of the weight matrix (W) with fixed gamma across layers. Consequently, it would be interesting to calculate the eigenvalues of the trained weights, check if there is a trend, and keep that trend in the minimal model. However, one could also argue that the gamma is already the effective timescale of a local population of neurons (here modeled by a rate code). What I want to aim at here is that in either case, it would be helpful that the results are related to the current concepts in theoretical neuroscience, possibly within the discussion.

- The claim "the network determinants of these timescale hierarchies are not well understood in realistic systems ... " is not well justified in the light of the above examples, specifically we recent new analyses such as (https://journals.plos.org/ploscompbiol/article?id=10.1371/journal.pcbi.1012355) or (https://www.pnas.org/doi/full/10.1073/pnas.2208998120) that look at timescales or eigenvalues along the hierarchy in models with noise.

- Mechanism of the presented results: Overall, I find the results very interesting and I believe consistent with my expectations from theoretical neuroscience. What I am lacking is more insight into how the results come about. The organization proceeds by introducing a model, showing a Figure (very nice consistent layout! I would suggest, however, to fix all x and y scales to the same range) and then relating that to Fig. 1. However, a mechanism why the model agrees more or less is not provided. I understand that this cannot be done extensively, but simple checks like Eigenvalues of the recurrent interactions or relation to the timescales of the leak would have been nice.

- Model parameter: I wonder if it helps to directly introduce timescales instead of the parameters gamma, alpha. Specifically Eq. (7) can be related to an exponential integration with a given timescale. This would possibly allow to explain increases in the response timescales "trivially".

- Models: I think I am lacking one detail about the model. Specifically, I fail to see what $x_t$ is for higher areas. Naively, I would have expected it to be $r_t$ (the nonlinear version of $h_t$) but this is the recurrent part. From the figure, it seems like h is passed up, but why is there no nonlinearity applied then. Also, why would it be $x_t$ and not $x_{t-1}$. I guess this is a misunderstanding on my side that originates from presenting the model very well in a simplified manner and may be clarified easily.

- Noise: In the model section, we define $\sigma = 0.7*\alpha$ which introduces two issues: i) why would we do that and not just change $\sigma$ and ii) there is an overlap with the $\boldsymbol\alpha$ from Table 6. Maybe sticking to $\sigma$ would be sufficient unless the 0.7 changes across layers (which I would not expect from the main text).

- Stimuli: The phase-scrambled movies I assume were obtained from shuffling the frequencies in Fourier space, but the sentence in line 370 does not tell us.

Reviewer #3: In this study, the authors investigate the mechanism required to explain the hierarchy of neural timescales observed in the rat brain. For this purpose, they use a previously developed CNN models of the ventral stream that integrates convolution and recurrence in its architecture and show that the implemented spatial invariance in this model is not sufficient to capture the hierarchy of timescales. Then, they show that including leaky integration and adaptation can introduce a hierarchical structure in network dynamics.

The study is interesting, and the details are presented clearly. However, I have several major comments regarding the main claims of the study listed below.

1. Biological plausibility of CORnet-RT and generalizability of the results:

CORnet-RT indeed has a higher brainscore than conventional CNNs, but the brainscore evaluates the time-averaged neural responses and not actual dynamics. The fact that the baseline CORnet-RT does not show a hierarchy of timescales and it is required to add more biological mechanisms, such as leaky integration and adaptation, is not surprising. Therefore, the claim that “intrinsic timescales depend on the details of the functions implemented on each layer” is very specific to this model. Additionally, stating that “the network determinants of these timescale hierarchies are not understood in realistic systems, as the classical theory is based on models without noise, recurrence, or adaptive mechanisms” is not true, considering the extensive literature of previous models built to explain the hierarchy of timescales. In fact, more biologically realistic models of primate and rodent brain that include noise and recurrence show that incorporating simple biological gradients is sufficient to capture the hierarchy of intrinsic timescales (Chaudhuri et al., 2015, Neuron) and their modulation during task (Wang et al., 2024, arXiv; Ding et al., 2024, eLife), without explicit task training. Therefore, it is important to avoid general claims that might be limited to this model, especially in the abstract. The authors also need to discuss their findings in the context of these prior studies, as well as the biological relevance of the mechanisms they propose.

2. Intrinsic timescales:

- Definition: I was quite surprised that intrinsic timescales measured from the rat data (Fig 1b) depend on the stimulus timescale, especially given the definition provided in the manuscript: “intrinsic timescales reflect the duration over which neural activity remains temporally correlated in the absence of stimulus variability”. If the timescales do depend on stimulus features, the term “intrinsic” is not appropriate, especially when it is measured from stimulus-driven activity.

- Estimation: For estimating intrinsic timescales from the CORnet-RT model, it would be more reasonable to use an uncorrelated noisy input (mimicking spontaneous neural activity) instead of movies. This is particularly important given the fact that in some figures (e.g., Leaky CORnet in Fig 4), a hierarchy of timescales is observed for some stimulus timescales and not others, and the authors have currently discarded these results.

3. Estimation of timescales from the model:

- The details of timescale estimation from CORnet-RT activity need to be clarified further in the methods. Given that CORnet-RT dynamics evolve on discrete time steps, how were they compared to spiking activity in seconds? How is the time discretization step defined, and how does it affect the results?

- It seems that CORnet-RT dynamics show an oscillatory component (based on the fitted function to autocorrelations). It would be interesting to report how the frequency of these oscillations changes across layers/models and also show some example autocorrelations of their behavior. Also, do authors observe a similar oscillation in neural activity?

3. Dependence of results on hyperparameters:

Models with leak and adaptation require a set of hyperparameters that seem to be manually chosen. It is important to show how the presented results depend on the choice of hyperparameters (γ, α, β).

4. Given the computational nature of this study, it is important that the code for the model simulations and analyses be made available for the review process (and after publication to other readers for reproducibility).

Minor comments:

1. Some references worth mentioning:

- Papers that also studied hierarchy of timescales in ANNs and their mechanisms that need to be discussed: Jain et al., 2020, NeurIPS; Moro et al., 2024, arXiv

- For lines 298-330, authors can cite Song et al., 2024, PNAS, which shows the hierarchy of intrinsic and response timescales is preserved across species.

2. What is the logic behind resampling a subset of neurons for computing timescales? To show the variability, it would have been more reasonable to train CORnet-RT with multiple seeds and perform analyses across multiple trained networks (but this might be beyond the scope of this study).

3. The role and location of nonlinearity in different models need to be clarified in the manuscript. For example, for the Minimal Model, where r_t is not used in h_t equation (eq. 10, 11), how does nonlinearity affect the dynamics? Does it affect transition of activity between layers?

Chaudhuri et al., 2015, Neuron https://doi.org/10.1016/j.neuron.2015.09.008

Ding et al., 2024, eLife https://doi.org/10.7554/eLife.85442

Wang et al., 2024, arXiv https://doi.org/10.1101/2023.06.04.543639

Jain et al., 2020, NeurIPS https://proceedings.neurips.cc/paper_files/paper/2020/hash/9e9a30b74c49d07d8150c8c83b1ccf07-Abstract.html

Moro et al., 2024, arXiv https://doi.org/10.48550/arXiv.2407.18838

Song et al., 2024, PNAS https://doi.org/10.1073/pnas.2415695121

**********

**Have the authors made all data and (if applicable) computational code underlying the findings in their manuscript fully available?**

The PLOS Data policy requires authors to make all data and code underlying the findings described in their manuscript fully available without restriction, with rare exception (please refer to the Data Availability Statement in the manuscript PDF file). The data and code should be provided as part of the manuscript or its supporting information, or deposited to a public repository. For example, in addition to summary statistics, the data points behind means, medians and variance measures should be available. If there are restrictions on publicly sharing data or code —e.g. participant privacy or use of data from a third party—those must be specified.

Reviewer #1: None

Reviewer #2: Yes

Reviewer #3: **No:** It is stated: The code used to generate the results and figures in this paper will be made available upon request.

**Figure resubmission:**

After uploading your figures to PLOS’s NAAS tool - https://ngplosjournals.pagemajik.ai/artanalysis, NAAS will process the files provided and display the results in the "Uploaded Files" section of the page as the processing is complete. If the uploaded figures meet our requirements (or NAAS is able to fix the files to meet our requirements), the figure will be marked as "fixed" above. If NAAS is unable to fix the files, a red "failed" label will appear above. When NAAS has confirmed that the figure files meet our requirements, please download the file via the download option, and include these NAAS processed figure files when submitting your revised manuscript.

**Reproducibility:**

To enhance the reproducibility of your results, we recommend that authors of applicable studies deposit laboratory protocols in protocols.io, where a protocol can be assigned its own identifier (DOI) such that it can be cited independently in the future. Additionally, PLOS ONE offers an option to publish peer-reviewed clinical study protocols. Read more information on sharing protocols at https://plos.org/protocols?utm_medium=editorial-email&utm_source=authorletters&utm_campaign=protocols

# Author comment: Author response to Decision Letter 1

**DOI:** 10.1371/journal.pcbi.1014653.r002

14 Jul 2026

Attachment Submitted filename: varetti_response_to_reviewers.pdf

# Peer review: Decision Letter 1

**DOI:** 10.1371/journal.pcbi.1014653.r003

**Authors:** Tianming Yang, Yuanning Li

3 Aug 2026

Dear Dr Piasini,

We are pleased to inform you that your manuscript 'Noisy models of the ventral stream reveal the impact of recurrence and learned representations on information processing timescales' has been provisionally accepted for publication in PLOS Computational Biology.

Before your manuscript can be formally accepted you will need to complete some formatting changes, which you will receive in a follow up email. A member of our team will be in touch with a set of requests.

Please note that your manuscript will not be scheduled for publication until you have made the required changes, so a swift response is appreciated.

IMPORTANT: The editorial review process is now complete. PLOS will only permit corrections to spelling, formatting or significant scientific errors from this point onwards. Requests for major changes, or any which affect the scientific understanding of your work, will cause delays to the publication date of your manuscript.

Should you, your institution's press office or the journal office choose to press release your paper, you will automatically be opted out of early publication. We ask that you notify us now if you or your institution is planning to press release the article. All press must be co-ordinated with PLOS.

Thank you again for supporting Open Access publishing; we are looking forward to publishing your work in PLOS Computational Biology.

Best regards,

Yuanning Li

Academic Editor

PLOS Computational Biology

Tianming Yang

Section Editor

PLOS Computational Biology

***********************************************************

Reviewer's Responses to Questions

**Comments to the Authors:**

**Please note here if the review is uploaded as an attachment.**

Reviewer #1: The authors have nicely addressed all of my concerns. Congratulations!

Reviewer #3: I thank the authors for their thorough rebuttal and detailed clarifications. They have addressed all my previous concerns and questions.

**********

**Have the authors made all data and (if applicable) computational code underlying the findings in their manuscript fully available?**

The PLOS Data policy requires authors to make all data and code underlying the findings described in their manuscript fully available without restriction, with rare exception (please refer to the Data Availability Statement in the manuscript PDF file). The data and code should be provided as part of the manuscript or its supporting information, or deposited to a public repository. For example, in addition to summary statistics, the data points behind means, medians and variance measures should be available. If there are restrictions on publicly sharing data or code —e.g. participant privacy or use of data from a third party—those must be specified.

Reviewer #1: None

Reviewer #3: Yes

**********

PLOS authors have the option to publish the peer review history of their article (what does this mean?). If published, this will include your full peer review and any attached files.

If you choose “no”, your identity will remain anonymous but your review may still be made public.

**Do you want your identity to be public for this peer review?**  For information about this choice, including consent withdrawal, please see our Privacy Policy.

Reviewer #1: No

Reviewer #3: No

# Editor report: Acceptance letter

**DOI:** 10.1371/journal.pcbi.1014653.r004

**Authors:** Tianming Yang, Yuanning Li

PCOMPBIOL-D-25-02544R1

Noisy models of the ventral stream reveal the impact of recurrence and learned representations on information processing timescales

Dear Dr Piasini,

I am pleased to inform you that your manuscript has been formally accepted for publication in PLOS Computational Biology. Your manuscript is now with our production department and you will be notified of the publication date in due course.

The corresponding author will soon be receiving a typeset proof for review, to ensure errors have not been introduced during production. Please review the PDF proof of your manuscript carefully, as this is the last chance to correct any errors. Please note that major changes, or those which affect the scientific understanding of the work, will likely cause delays to the publication date of your manuscript.

Soon after your final files are uploaded, unless you have opted out, the early version of your manuscript will be published online. The date of the early version will be your article's publication date. The final article will be published to the same URL, and all versions of the paper will be accessible to readers.

For Research, Software, and Methods articles, you will receive an invoice from PLOS for your publication fee after your manuscript has reached the completed accept phase. If you receive an email requesting payment before acceptance or for any other service, this may be a phishing scheme. Learn how to identify phishing emails and protect your accounts at https://explore.plos.org/phishing.

Thank you again for supporting PLOS Computational Biology and open-access publishing. We are looking forward to publishing your work!

With kind regards,

Janani Seenivasan

PLOS Computational Biology | Carlyle House, Carlyle Road, Cambridge CB4 3DN | United Kingdom ploscompbiol@plos.org | Phone +44 (0) 1223-442824 | ploscompbiol.org | @PLOSCompBiol
